# Peritoneal Cells Mediate Immune Responses and Cross-Protection Against Influenza A Virus

**DOI:** 10.3389/fimmu.2019.01160

**Published:** 2019-05-28

**Authors:** Avishekh Gautam, Byoung Kwon Park, Te Ha Kim, Madhav Akauliya, Dongbum Kim, Sony Maharjan, Joongwon Park, Jinsoo Kim, Hanseul Lee, Man-Seong Park, Younghee Lee, Hyung-Joo Kwon

**Affiliations:** ^1^Department of Microbiology, College of Medicine, Hallym University, Chuncheon, South Korea; ^2^Center for Medical Science Research, College of Medicine, Hallym University, Chuncheon, South Korea; ^3^Department of Microbiology, College of Medicine, and the Institute for Viral Diseases, Korea University, Seoul, South Korea; ^4^Department of Biochemistry, College of Natural Sciences, Chungbuk National University, Cheongju, South Korea

**Keywords:** apoptosis, cross protection, influenza A virus, neutralizing antibody, peritoneal cells

## Abstract

Intraperitoneal inoculation with live influenza A virus confers protection against intranasal infections in mice and ferrets. However, the responses of peritoneal cells to influenza A virus have not been investigated. Here we show that intraperitoneal inoculation with A/WSN/1933 (H1N1) virus induced virus-reactive IgG production in the peritoneal cavity in mice. The infection resulted in substantial but transient B cell and macrophage depletion along with massive neutrophil infiltration, but virus growth was not detected. Influenza A viruses bound to α-2,6-linked sialic acids of B cells and macrophages and induced apoptotic death of peritoneal cavity cells. However, re-infection with A/WSN/1933 virus did not have adverse effects on immune cells most likely because of the neutralizing antibodies produced in response to the first exposure. Infection of BALB/c mice with A/WSN/1933 induced cross-protection against an otherwise lethal intraperitoneal dose of A/Hongkong/4801/2014 (H3N2) virus. This information suggests that immunological responses in the peritoneal cavity can induce effective defense against future virus infection. Considering the unexpected potent immunoregulatory activity of the peritoneal cells against influenza viruses, we suggest that comparative studies on various immune reactions after infection through different routes may contribute to better selection of vaccination routes in development of efficacious influenza vaccines.

## Introduction

Influenza viruses are one of the most significant causes of morbidity and mortality among all respiratory tract infections. Frequent endemic and pandemic outbreaks of influenza A virus infection have claimed thousands of lives, mostly those of infants, the elderly and immunosuppressed individuals (1–3). The presence of a segmented genome allows the virus to undergo reassortment, resulting in genotypically and phenotypically different subtypes and the rapid variation poses a notoriously difficult obstacle to sustainable vaccine development.

For successful development of vaccines targeting influenza A virus, a better understanding of the immune responses in the early stage of vaccination and optimization of the vaccination protocol based on the information is required. In particular, there can be immunological differences depending on the route of vaccination (4–9). Current human influenza vaccines are administered intramuscularly or intranasally. Research on intranasal administration has gained attention recently due to effective induction of protective immunity by triggering mucosal responses (10–12). However, research on influenza vaccine development in mice has preferentially utilized the intraperitoneal route of immunization because of the experimental convenience and empirical effectiveness (13–15). In fact, intraperitoneal inoculation of live influenza virus has been shown to confer protection against intranasal infections in mice and ferrets (16–19).

Cellular and immune responses to intranasal infection of influenza virus have been studied (20–23). Intranasal infection of mice with influenza A virus induces pulmonary disease and results in an impaired immune system, with effects such as severe depletion of blood lymphocytes, bone marrow cells, and lung B cells (20–23). Influenza A virus has been shown to not be directly involved in the attrition of lymphocytes, but virus-induced cytokines such as tumor necrosis factor-α (TNF-α) and lymphotoxin-α (LT-α) are crucial in this phenomenon (20). The interaction of the B cell receptor with hemagglutinin (HA) has been shown to induce depletion of B cells in the lung after influenza A virus infection (23). Additionally, the hypothalamic–pituitary axis and sympathetic nervous responses were credited with the substantial loss of B cells upon infection with the H9N2 avian influenza virus (24). However, the response of peritoneal cells to intraperitoneal inoculation of influenza A virus has not been investigated. Therefore, investigating the immune response against influenza A virus infection in the peritoneal cavity in mice could provide novel information that might aid in human influenza vaccine development.

Here, we have studied the immune response of peritoneal cells to influenza A virus infection using the BALB/c mouse model, which expresses both α-2,3-linked and α-2,6-linked sialic acid receptors (25–27) essential for influenza A virus binding to epithelial cells. We used a mouse-adapted influenza A virus strain A/WSN/1933 (H1N1, WSN) in this study and found that intraperitoneal inoculation of A/WSN/1933 virus modulated immune cell populations and induced robust production of influenza A virus-reactive antibody. Furthermore, we observed that intraperitoneal inoculation with A/WSN/1933 virus induced a protective effect against lethal A/Hongkong/4801/2014 (H3N2) exposure. These results suggest that immunological responses in the peritoneal cavity are crucially effective upon influenza A virus infection. This information might be very useful for future development of effective vaccines.

## Materials and Methods

### Cell Line and Virus

Madin-Darby canine kidney (MDCK) cell lines were purchased from American Type Culture Collection (ATCC, Manassas, VA, USA) and maintained in minimum essential medium (MEM) containing 10% fetal bovine serum (FBS) and antibiotics (100 μg/ml streptomycin and 100 U/ml penicillin). The influenza virus strains used in this experiment are A/WSN/1933 (WSN, mouse adapted H1N1), A/Hongkong/4801/2014 (non-mouse-adapted H3N2), rIETR CVV (H5N1), and modified NIBRG-268M (H7N9). rIETR candidate vaccine virus (CVV) (H5N1) with low pathogenic HA and NA genes from A/chicken/Korea/IS/2006 (IS06; a highly pathogenic avian influenza H5N1 virus isolated in Korea, clade 2.2) and internal genes from A/PR/9/34(H1N1) virus was generated by reverse genetics (28). NIBRG-268M (H7N9) was generated by reverse genetics based on NIBRG-268 candidate vaccine virus with HA and NA genes from A/Anhui/1/2013 (H7N9) and internal genes from PR8 backbone virus. Viruses were produced either by inoculation into specific-pathogen-free (SPF) embryonated chicken eggs or by infecting MDCK cell lines. Biosafety level 2 conditions were strictly maintained during the virus preparation and cell culture procedure.

### Plaque Assay

Viruses were quantified by a plaque assay. MDCK cells were seeded at 7 × 10^5^ cells per well in six-well plates and incubated at 37 °C. After culturing to a confluent monolayer, the cells were washed twice with PBS and infected with tenfold serial dilutions of influenza virus suspension. Following 1 h incubation with shaking at 15-20 min intervals, the suspensions were removed by suction, and the cells were overlaid with 2 ml of DMEM/F12 media containing 2 mM glutamine, 4% BSA, 10 mM HEPES, 2.5% sodium bicarbonate, 50 mg/ml DEAE dextran, 1 μg/ml L-tosylamide-2-phenylethyl chloromethyl ketone (TPCK)-treated trypsin, 100 U/ml penicillin, 100 μg/ml streptomycin and 0.6% immunodiffusion-grade agar. After 72 h incubation at 37 °C, the plates were stained with crystal violet (0.1% crystal violet in 20% methanol) for 1 h. Plaques were enumerated, and the virus titers were determined.

### Virus Inactivation by Ultraviolet Irradiation

A/WSN/1933 virus was inactivated by exposure to 254 nm UV light with 1,500 mM/s/cm^2^ UV for 15 min from a height of 5 cm. Inactivation was confirmed by a plaque assay as described previously (29). The absence of any visible plaques assured virus inactivation.

### Ethics Statement

All animal procedures performed in this study are in accordance with the recommendations in the Guide for the Care and Use of Laboratory Animals of the National Veterinary Research & Quarantine Service of Korea. This study was approved by the Institutional Animal Care and Use Committee of Hallym University (Permit Number: Hallym2015-54 and 2017-41). Mice were anesthetized by 1-2% isoflurane inhalation (JW Pharmaceutical, Seoul, Korea) to minimize pain. Mice were euthanized by CO_2_ inhalation if they lost 25% of their baseline adult body weight or if they revealed evidence of debilitation, pain or distress such as hunched posture, rough hair coat, reduced food consumption, emaciation, inactivity, ambulation difficulty, and respiratory problems. After the experiments were terminated, the mice were sacrificed by CO_2_ inhalation, and all efforts were made to minimize suffering.

### Mice and Infection of Virus

Eight-week-old BALB/c (H-2^b^) mice were purchased from Nara Biotech, Inc. (Seoul, Korea) and maintained under SPF conditions. All virus infection animal experiments were carried out under stringent animal biosafety level 2 conditions. A/WSN/1933 virus or UV-inactivated A/WSN/1933 (UV-WSN) was intraperitoneally infected at a dose of 5 × 10^6^ pfu per mouse. When necessary, the infected mice were reinfected intraperitoneally with A/WSN/1933 virus at a dose of 5 × 10^6^ pfu per mouse 5, 7, or 14 days after the first infection.

### ELISA

Ninety-six-well immunoplates (Nunc^TM^, Roskilde, Denmark) were coated with influenza A viruses for quantitation of influenza A virus-reactive IgG or IgM, respectively. The coated plates were incubated overnight at 4°C. The plates were then washed three times with PBST (0.1% Tween-20 in PBS) and blocked with 1% BSA for 1 h. The plates were loaded with threefold dilutions of sera or peritoneal cavity fluids in PBST and then incubated for 2 h at room temperature. After the plates were washed three times with PBST, horseradish peroxidase (HRP)-labeled goat anti-mouse IgG/IgG1/IgG2a/IgG2b/IgG3/IgM antibodies (Catalog No: 5300-05, Southern Biotechnology Associates, Inc., Birmingham, AL, USA) (1:500 dilution) were used for the detection of total or subclass- and isotype-specific antibodies. After incubation for 1 h at room temperature, the plates were washed three times with PBST, and colorimetrically developed using TMB (3,3′,5,5′-tetramethylbenzidine) substrate solution (Kirkegaard and Perry Laboratories, Gaithersburg, MD, USA). Absorbance was measured using a SpectraMax 250 microplate reader (Molecular Devices, Sunnyvale, CA, USA) at 450 nm.

### Analysis of Cell Populations

Mice were anesthetized, sacrificed, and peritoneal cells, splenocytes and bone marrow cells were harvested using RPMI 1640 media containing 5% FBS as described previously (30, 31). Cells were centrifuged at 1,200 rpm for 5 min. The peritoneal cavity fluids were collected for ELISA. Bone marrow cells and splenocytes were treated with 5 ml RBC lysis buffer (20 mM Tris HCl, 140 mM NH_4_Cl) for 5 min. We pre-gated peritoneal cells, bone marrow cells, splenocytes (FSC^low^SSC^low^ or FSC^low^SSC^high^) for the enriched lymphoid and myeloid population and then checked specific cell population. Total cell numbers of peritoneal cavity, spleen, and bone marrow were counted and analyses of cell populations were performed as described elsewhere (32, 33). Cells were resuspended in RPMI and then washed with FACS buffer (1% FBS in PBS) and treated with 10 μg/ml anti-FcγRII/III antibody (Catalog No: 553142, BD Biosciences, San Jose, CA, USA) for 20 min at 4 °C to block the Fc receptors. Cells were then incubated with rat anti-mouse fluorescent antibodies to characterize the lymphoid and myeloid populations. For the lymphoid population, the antibodies used were PerCP Cy5.5-conjugated anti-CD3 (Catalog No: 551163, BD Biosciences), APC-conjugated anti-CD4 (Catalog No: 553051, BD Biosciences), FITC-conjugated anti-CD8 (Catalog No: 553031, BD Biosciences), FITC-conjugated anti-B220 (Catalog No: 553088, BD Biosciences), BV421-conjugated anti-CD19 (Catalog No: 562701, BD Biosciences), PE-conjugated anti-CD23 (Catalog No: 12-0232-81, eBioscience, San Diego, CA, USA). For the myeloid population, the antibodies used were PE-conjugated anti-CD11c (Catalog No: 557401, BD Biosciences), APC-conjugated anti-CD11b (Catalog No: 553312, BD Biosciences), APCeF780-conjugated anti-Ly6G (Catalog No: 47-5931-82, eBioscience), and FITC-conjugated anti-F4/80 (Catalog No: 11-4801-85, eBioscience). For the IgG^+^ and IgM^+^ population, the antibodies used were FITC-conjugated anti-IgM (Catalog No: 553437, BD Biosciences), Biotin-conjugated anti-IgG1 (Catalog No: 553441), APC-conjugated Streptavidin (Catalog No: SA1005, Thermo Fisher Scientific, Waltham, MA, USA). To exclude dead cells, cells were incubated with 7AAD (Catalog No: 51-68981E, BD Biosciences). After 1 h incubation at 4°C, the cells were washed with FACS buffer and analyzed with a FACSCanto^TM^ II (Becton Dickinson, Franklin Lakes, NJ, USA).

### Annexin V Staining

Peritoneal cells were harvested from BALB/c mice. Analyses of apoptosis were performed as described elsewhere (34). The cells were then plated in 24-well plates at a density of 1 × 10^6^ cells per well. If necessary, SNA (10 μg/ml) pretreatment was applied. A/WSN/1933 virus or UV-WSN virus was added to each well at a 1 × 10^6^ pfu. After incubation at 37°C for different time periods, cells were harvested and stained with anti-FcγRII/III (Fc receptor blocker) before being stained with APCef780-conjugated anti-CD19 (Catalog No: 47-0193-80, eBioscience), PE-conjugated anti-CD23 (Catalog No: 12-0232-81, eBioscience), FITC-conjugated anti-B220 (Catalog No: 553088, BD Biosciences), FITC-conjugated anti-F4/80 (eBioscience), and BV421-conjugated anti-CD11b antibodies (Catalog No: 48-0112-80; eBioscience). After 1 h incubation at 4 °C, cells were washed with FACS buffer and stained with APC-conjugated Annexin V (Catalog No: 17-8007-74, eBioscience) for 15 min at room temperature. To exclude dead cells, stained cells were incubated with 10 μg/mL of PI (eBioscience, San Diego, CA, USA) or 7AAD (Catalog No: 51-68981E, BD Biosciences). The cells were analyzed with a FACSCanto^TM^ II.

### Western Blotting

Peritoneal cells (1 × 10^6^ cells per well) were plated in 24-well plates. The cells were treated with SNA (10 μg/ml) and/or infected either with A/WSN/1933 virus or UV-WSN virus (1 × 10^6^ pfu) and incubated at 37 °C. At different time intervals, cells were harvested and lysed with Triton X-100 lysis buffer (10 mM HEPES, pH 7.4, 150 mM NaCl, 5 mM EDTA, 1% Triton X-100). The lysates were separated by 12% sodium dodecyl sulfate-polyacrylamide gel electrophoresis (SDS-PAGE), and the apoptotic proteins were analyzed by western blotting. We obtained rabbit polyclonal anti-PARP (Catalog No: 9542S, Cell Signaling Technology, Danvers, MA, USA) and anti-cleaved Caspase-3 (Catalog No: 9661S, Cell Signaling Technology) antibodies from Cell Signaling Technology, rabbit polyclonal anti-Bcl-2 antibody from Delta Biolabs (Catalog No: GTX100064, Campbell, CA, USA) and mouse anti-β-actin antibody from Sigma-Aldrich (Catalog No: A5316, St. Louis, MO, USA). Viral protein samples were prepared by mixing influenza A viruses with SDS sample buffer and boiling for 10 min. Gradient SDS-PAGE (4–12%) (Thermo Fisher Scientific) and western blotting analysis was carried out as mentioned elsewhere (35). Viral proteins were detected by western blotting using peritoneal cavity fluids (1:10 dilution in PBS), which were harvested from BALB/c mice after 14 days of A/WSN/1933 virus inoculation.

### Lectin Staining

Peritoneal cells were harvested from BALB/c mice and incubated with A/WSN/1933 virus or UV-WSN virus for 1 h at 4 °C. The cells were then stained for 1 h at 4°C with fluorescein-conjugated *Sambucus nigra* agglutinin (SNA), which was obtained from Vector Laboratories (Burlingame, CA, USA). The cells were analyzed by flow cytometry (BD FACSCalibur^TM^, BD Biosciences).

### Hemagglutination Inhibition (HI) Assay

Ninety-six-well V-bottom plates (Costar, Corning, NY, USA) were used for the HI assay. Peritoneal cavity fluids from PBS-injected or A/WSN/1933 virus-infected BALB/c mice were serially diluted two-fold with PBS and then incubated with an equal volume of 4 hemagglutination units (4HA) of each influenza A virus for 30 min. After incubation, an equal volume of 0.5% chicken red blood cells were added to the wells and incubated for 30 min at room temperature, and HI titers were measured.

### Virus Neutralization Assay

The peritoneal cavity fluids of A/WSN/1933 virus-infected BALB/c were serially diluted twofold with PBS and then incubated with approximately 100 pfu/ml of A/WSN/1933, A/Hongkong/4801/2014 (H3N2), rIETR CVV (H5N1), NIBRG-268M (H7N9) at 37°C for 1 h. The samples were added to a confluent monolayer of MDCK cells in MEM supplemented with 10% FBS and TPCK-treated trypsin, and a plaque assay was performed as described above. The neutralization percentage was measured by the following equation: neutralization (%, percent inhibition) = [(plaque number with virus only – plaque number with serially diluted peritoneal cavity fluids mixed with virus) / plaque number with virus only] x 100.

### Virus Superinfection

Eight-week-old BALB/c (H-2^b^) mice (*n* = 10) were injected intraperitoneally with A/WSN/1933 virus at a dose of 5 × 10^6^ pfu per mouse. After 7 days, the mice were intraperitoneally challenged with 1 × 10^8^ pfu of wt A/Hong Kong/4801/2014 (H3N2) virus, and then the mice were observed for 14 days to monitor their clinical signs and body weight. To analyze the cell population in the virus-infected mice, we prepared cells from the peritoneal cavity and bone marrow of the mice at 5 days after a single intraperitoneal challenge with 1 × 10^8^ pfu of H3N2 virus or from mice that were inoculated with A/WSN/1933 virus (5 × 10^6^ pfu) and then inoculated 7 days later with H3N2 virus (1 × 10^8^ pfu); 5 days after the second inoculation, the cells were stained with PerCP Cy5.5-conjugated anti-CD3, BV421-conjugated anti-CD19 and then analyzed with a FACSCanto^TM^ II.

### Statistical Analysis

The results are shown as the mean ± standard deviation. The statistical significance of differences between two samples was evaluated using Student's *t*-test; *P* < 0.05 was considered statistically significant.

## Results

### A/WSN/1933 Virus Efficiently Induces Antibody Production in the Peritoneal Cavity

**Figure 1:**
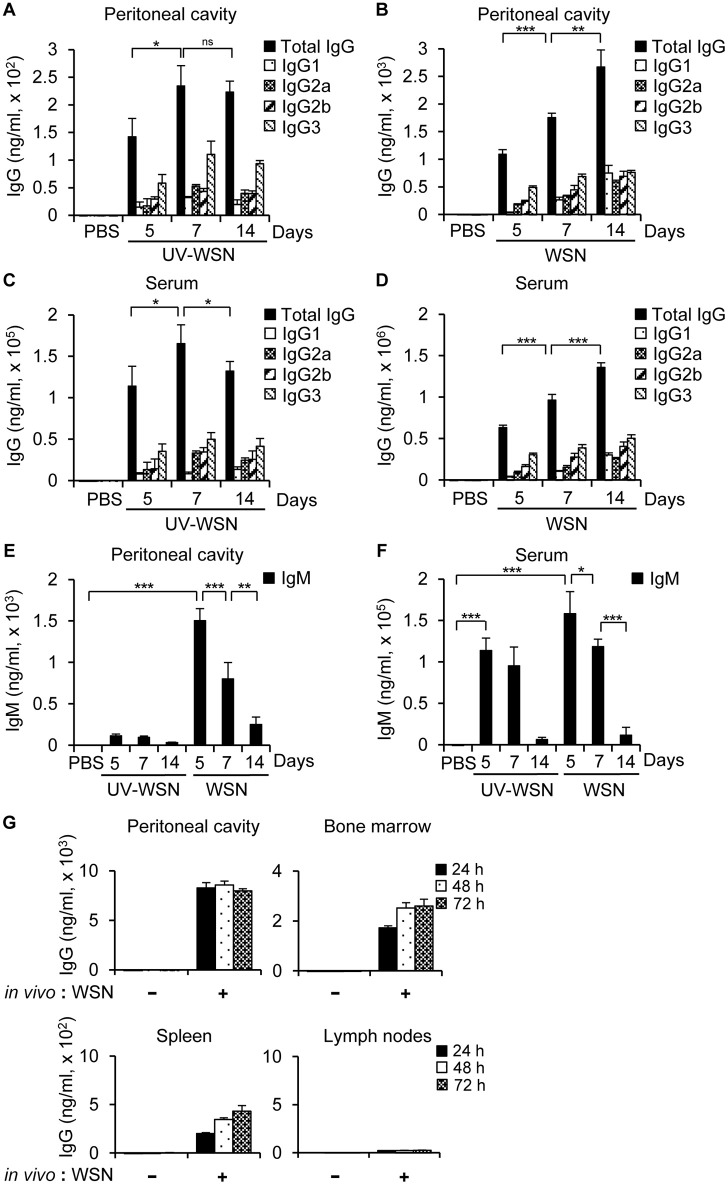
Antibody production in BALB/c mice following intraperitoneal challenge with A/WSN/1933 virus or UV-inactivated A/WSN/1933 virus. BALB/c mice (*n* = 5/group) were anesthetized at 5, 7, and 14 days after intraperitoneal challenge with 5 × 10^6^ pfu of live A/WSN/1933 virus (WSN) or UV-inactivated A/WSN/1933 virus (UV-WSN), and samples of peritoneal cavity fluid and serum were collected. **(A,B)** The amounts of A/WSN/1933 virus-reactive IgG and IgG subclasses in the peritoneal cavity fluids following UV-WSN virus **(A)** and A/WSN/1933 virus **(B)** challenge were quantified. Please note the different scales used for the y-axes of graphs **(A,B)**. **(C, D)** A/WSN/1933 virus-reactive IgG and IgG subclasses in the serum were quantified following UV-WSN virus **(C)** and A/WSN/1933 virus **(D)** challenge. Please note the different scales used for the y-axes of graphs **(C,D)**. **(E,F)** IgM concentrations in the peritoneal cavity fluids **(E)** and the serum **(F)** were measured. **(G)** Cells from the peritoneal cavity, bone marrow, spleen, and mesenteric lymph nodes (1 × 10^6^ cells) were obtained from BALB/c mice 14 days after intraperitoneal A/WSN/1933 virus infection (*in vivo*: WSN) and then cultured in RPMI 1640 medium containing 10% FBS and antibiotics for indicated time intervals. Antibody concentrations of the cell culture supernatants were determined by ELISA. These data are representative of three independent experiments. ns, not significant. ^*^p < 0.05, ^**^p < 0.005, ^***^p < 0.0005.

It was previously reported that the live A/WSN/1933 virus is more immunogenic and protective than the inactivated virus when administered intramuscularly (8). It was also proved in a research comparing live and inactivated A2/Hong Kong influenza A virus vaccines when administered intranasally (36). To clarify this issue in the peritoneal cavity, we first examined virus-induced antibody production. To this end, we inoculated BALB/c mice intraperitoneally with untreated A/WSN/1933 virus or UV-WSN virus and antibody production in the peritoneal cavity fluids was measured by ELISA on days 5, 7, and 14 post-infection. In contrast to A/WSN/1933 virus-infected mice that exhibited a steady increase in A/WSN/1933 virus-reactive IgG levels from 5 to 14 days post-infection in peritoneal cavity fluid (Figure 1B), A/WSN/1933 virus-reactive IgG levels in UV-WSN-infected mice increased from 5 to 7 days and then plateaued (Figure 1A) and the IgG production was virus-dosage dependent (Figure S1). In the serum of the UV-WSN virus-infected mice, A/WSN/1933 virus-reactive IgG levels increased until 7 days post-infection and then decreased at 14 days post-infection (Figure 1C). IgG levels in the serum of A/WSN/1933 virus-infected mice (Figure 1D) showed the same trend as in the peritoneal cavity fluids (Figure 1B). The levels of A/WSN/1933 virus-reactive IgM in the peritoneal cavity fluids (Figure 1E) and serum (Figure 1F) of A/WSN/1933 virus-infected mice decreased gradually from 5 to 14 days post infection. The same tendency was also found in the UV-WSN virus-infected mice. The concentrations of A/WSN/1933 virus-reactive IgG in both serum and peritoneal cavity fluids of A/WSN/1933 virus-infected mice were markedly higher than those of UV-WSN virus-infected mice (The scale of A/WSN/1933 data is tenfold higher than UV-WSN data in Figures 1A–D). The levels of A/WSN/1933 virus-reactive IgM in the peritoneal cavity were also substantially higher in A/WSN/1933 virus-infected mice than those of UV-WSN virus-infected mice (Figure 1E). However, the difference of IgM concentrations was much milder in the serum (Figure 1F). Robust production at early time points and followed decrease of A/WSN/1933 virus-reactive IgM and consistent increase of A/WSN/1933 virus-reactive IgG over time suggests class switching of B cells producing virus-reactive antibodies. Taken together, these results show that A/WSN/1933 virus is more efficient than UV-WSN virus in inducing A/WSN/1933 virus-reactive antibody production in the peritoneal cavity and serum when administered intraperitoneally.

To identify the cellular origin of influenza A virus-reactive antibodies, cells from the peritoneal cavity, bone marrow, spleen, and mesenteric lymph nodes of mice were collected 14 days after intraperitoneal inoculation with PBS or A/WSN/1933 virus and cultured *in vitro*. Culture supernatants were separated at different time points and ELISA were carried out to determine the quantity of IgG. We observed that A/WSN/1933 virus-reactive antibody was produced by cells of the peritoneal cavity, bone marrow and spleen, but not by cells of the mesenteric lymph nodes *in vitro*.

### Lymphocyte Populations Exhibit Changes Following Intraperitoneal Challenge With A/WSN/1933 Virus

**Figure 2:**
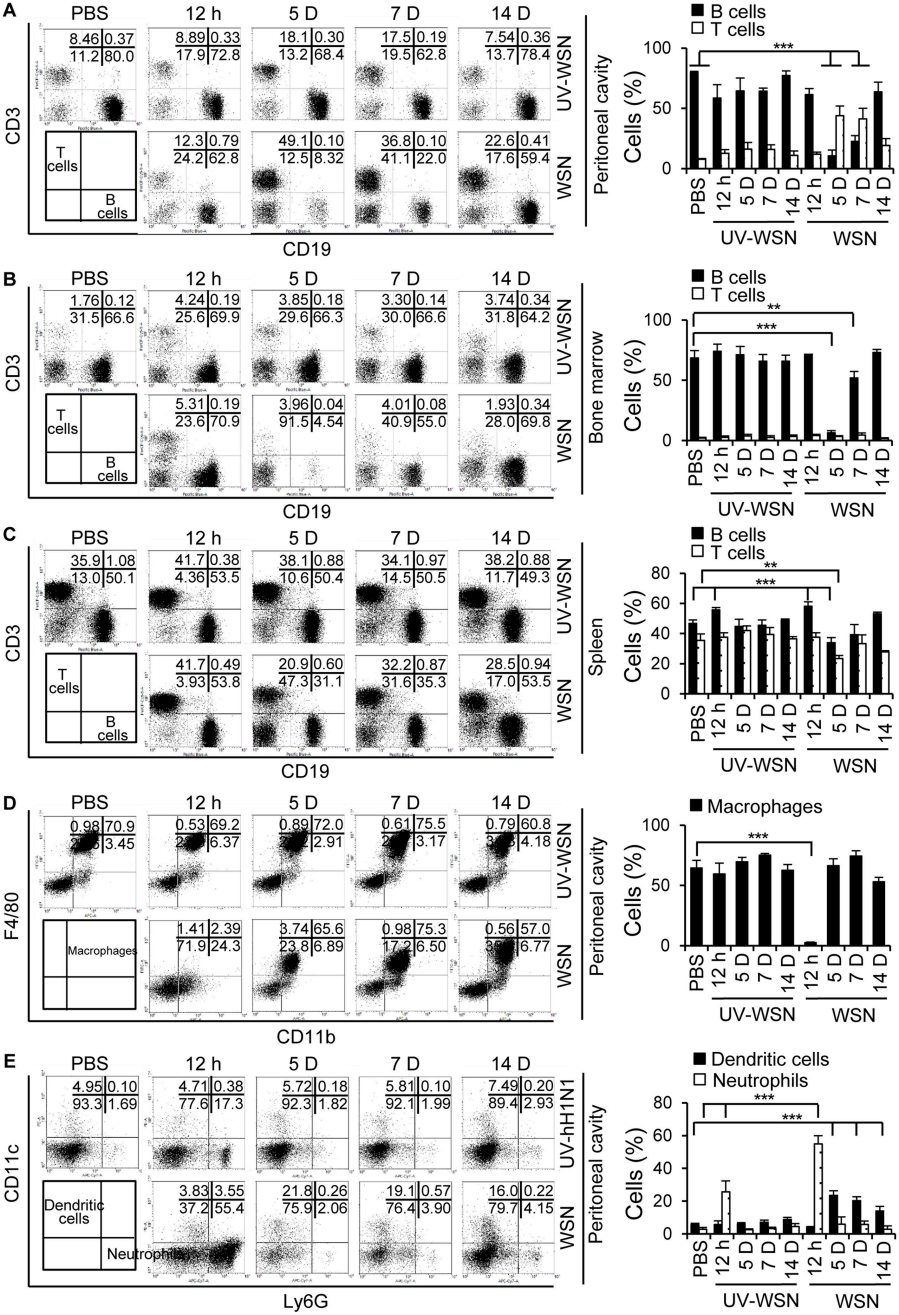
Analysis of cell populations following intraperitoneal challenge with A/WSN/1933 virus or UV-inactivated A/WSN/1933 virus. BALB/c mice (*n* = 5/group) were sacrificed at 12 h, 5, 7, and 14 days after intraperitoneal challenge with 5 × 10^6^ pfu of A/WSN/1933 virus (WSN) or UV-inactivated A/WSN/1933 virus (UV-WSN). Peritoneal cells, bone marrow cells and splenocytes were harvested, counted, and stained with fluorescence-conjugated antibodies and analyzed by flow cytometry. Cells from control mice at 14 days after intraperitoneal injection of PBS were used as an uninfected control. **(A-C)** FSC^low^SSC^low^ cells of peritoneal cells, bone marrow cells, splenocytes were gated, and lymphocyte populations of the peritoneal cavity **(A)**, bone marrow **(B)**, and spleen **(C)** were sorted into CD3 and CD19 subsets. **(D,E)** Peritoneal cells (FSC^low^SSC^high^) were gated, and two populations, F4/80^high^CD11b^high^ (macrophages) and F4/80^low^CD11b^low^ (non-macrophages) were analyzed. **(D)** Peritoneal macrophages (F4/80^high^CD11b^high^) were sorted for F4/80 and CD11b double-positive phenotype. **(E)** The dendritic cell and neutrophil populations of the peritoneal cavity were sorted from F4/80^low^CD11b^low^ population using CD11c and Ly6G markers. Right panel showed the indicated cell population (%) among FSC^low^SSC^low^ cells (A-C) and FSC^low^SSC^high^ cells **(D,E)**. Each figure is representative of three independent experiments. ^**^p < 0.005, ^***^p < 0.0005.

To analyze any changes in lymphocyte cell populations following intraperitoneal inoculation of BALB/c mice with A/WSN/1933 virus or UV-WSN virus, we performed flow cytometric analysis on peritoneal cells, bone marrow cells and splenocytes. We sorted the lymphocyte populations (FSC^low^SSC^low^) into CD3- and CD19-expressing subpopulations. Following intraperitoneal inoculation with A/WSN/1933 virus, a 7-fold and 15-fold depletion of B cells (CD19 positive populations) was observed at 5 days post-infection in the peritoneal cavity (Figure 2A, Figure S2A) and bone marrow (Figure 2B, Figure S2B), followed by partial recovery at 7 days and restoration to near-normal levels at 14 days post-infection in bone marrow. In the peritoneal cavity, increase in the numbers of B cells (1.4 fold) and B-1 cells (1.4 fold) was observed at 14 days post-infection compared to normal mice (Figures S2A, S3A). Furthermore, IgG^+^ B-1 cells and IgG^+^ B-2 cells were increased at 14 days post-infection (Figure S4). Notably, B-1 cell population was the major population of B cells in the peritoneal cavity and bone marrow during the experimental periods (Figures S3, S4).

The peritoneal cavity was heavily infiltrated with T cells (CD3-positive populations) at 5 days post-infection, and CD3-positive populations slowly declined toward normal values at 14 days post-infection (Figure 2A). Total cell numbers of T cells, CD4^+^ and CD8^+^ T cells increased at 5 to 14 days post-infection in the peritoneal cavity (Figures S2A, S5). Splenic B cells displayed a transient decrease at 5 days post-infection; the extent of B cell reduction in levels was much less severe in comparison to peritoneal and bone marrow B cells (Figure 2C, Figure S2C). We also noted an apparent reduction in splenic T cells at 5 days post-infection (Figure 2C, Figure S2C). Additionally, macrophage populations (F4/80 and CD11b double positive) in the peritoneal cavity were nearly completely depleted at 12 h post-infection and partially recovered at 5 days with gradually restoration to normal levels by 14 days (Figure 2D, Figure S2A). Peritoneal neutrophils populations (Ly6G-positive), on the other hand, remarkably increased 12 h after A/WSN/1933 virus infection and returned to normal levels at 5 days (Figure 2E, Figure S2A). CD11c-positive dendritic cell populations increased at 5 days and then gradually decreased in the peritoneal cavity of A/WSN/1933 virus-infected mice (Figure 2E). UV-WSN virus inoculation had little or no effect on lymphocyte populations. Interestingly, we did not observe any mortality or morbidity in the mice intraperitoneally infected with live or UV-irradiated virus, suggesting that the altered lymphocyte and macrophage populations were not associated with any overt adverse effects at the time points studied.

### Cells of the Peritoneal Cavity Undergo Apoptosis Following A/WSN/1933 Virus Inoculation

**Figure 3:**
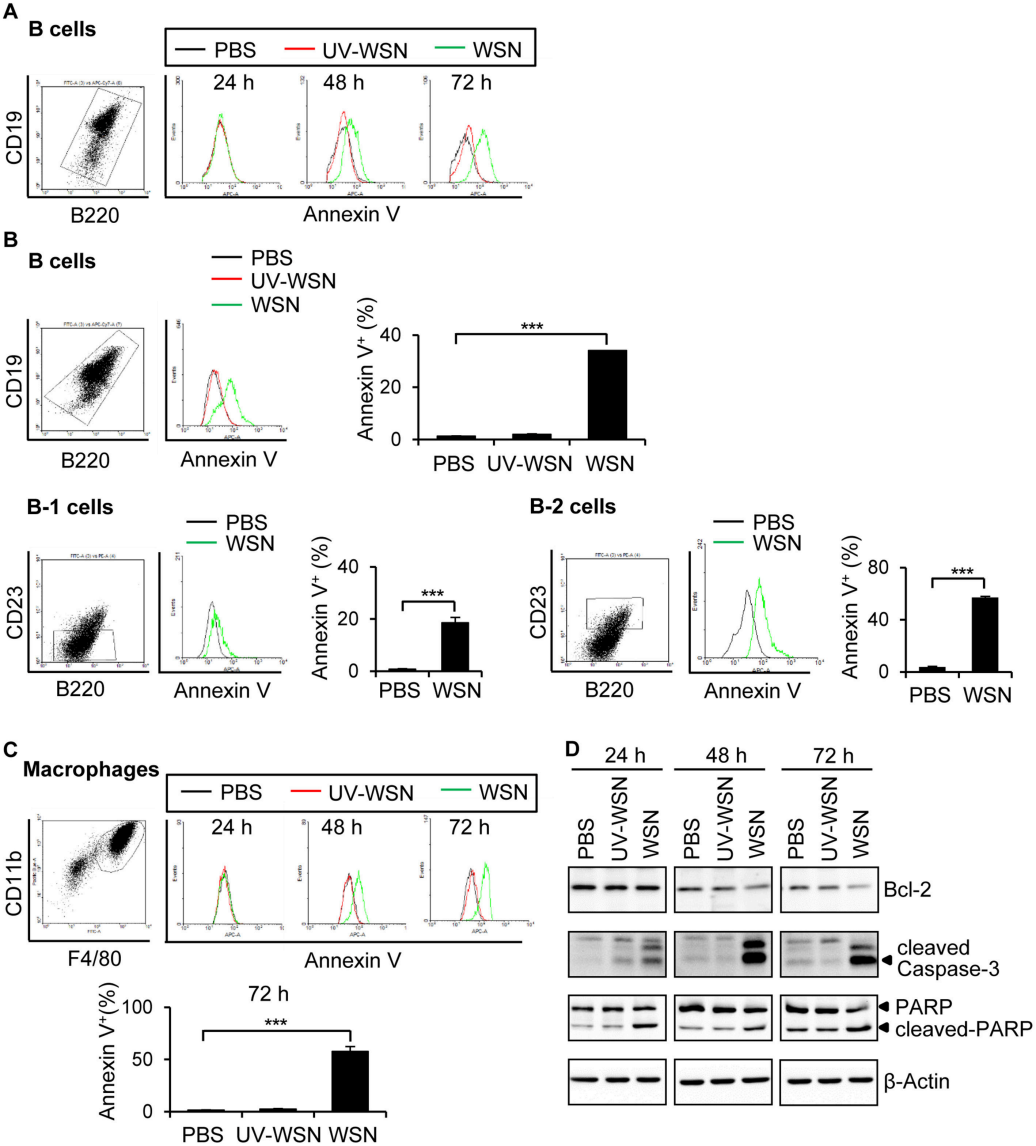
Peritoneal cells undergo apoptosis after A/WSN/1933 virus infection. **(A)** BALB/c mice (*n* = 3/group) were sacrificed, and peritoneal cells were harvested. The cells (1 × 10^6^) were seeded into 24-well plates containing RPMI 1640 complete media, and then treated with 1 × 10^6^ pfu of A/WSN/1933 virus (WSN) or UV-WSN virus (UV-WSN). After incubation for 24, 48, or 72 h, the cells were stained with fluorescence-conjugated antibodies (CD19, B220) and Annexin V. After washing, PI detection kit was used to discard dead cells and then analyzed by flow cytometry. Annexin V-positive B cells are represented in histograms. **(B)** Peritoneal cells (1 × 10^6^) were treated with 1 x 10^6^ pfu of A/WSN/1933 virus (WSN) or UV-WSN virus (UV-WSN) for 72 h, the cells were stained with fluorescence-conjugated antibodies (CD19, B220, CD23, 7AAD) and Annexin V. Annexin V-positive B cells are represented in histograms. Each right panel of **(B)** shows the % of annexin V-positive B cells, B-1 cells, and B-2 cells. **(C)** Peritoneal cells (1 × 10^6^) were treated with 1 x 10^6^ pfu of A/WSN/1933 virus (WSN) or UV-WSN virus (UV-WSN). After incubation for 24, 48, or 72 h, the cells were stained with fluorescence-conjugated antibodies (CD11b, F4/80) and Annexin V. After washing, PI detection kit was used to discard dead cells and then analyzed by flow cytometry. Annexin V-positive macrophages are represented in histograms. Lower panel of **(C)** shows the % of annexin V-positive macrophages. **(D)** Time-dependent expression of Bcl-2, cleaved Caspase-3 and cleaved PARP was determined by western blotting. Each figure is representative of three independent experiments. ^***^P < 0.0005.

To investigate whether the B cells and macrophages depletion observed in mice following A/WSN/1933 virus infection was due to apoptosis, we cultured peritoneal cells harvested from untreated BALB/c mice in the presence of PBS, A/WSN/1933 virus, or UV-WSN virus and stained them with Annexin V that binds to phosphatidylserine, a marker of apoptosis. While *in vitro* infection with A/WSN/1933 virus induced a time-dependent and dosage-dependent increase in Annexin V-stained populations of B cells (Figures 3A,B, Figure S6A) and macrophages (Figure 3C, Figure S6B), infection with UV-WSN virus did not induce any changes in the numbers of Annexin V-stained peritoneal cells (Figures 3A–C). As shown in Figure 3B, about 37% of B cells were subjected to apoptosis in response to A/WSN/1933 virus infection. About 20 % of B-1 cells and 50% of B-2 cells were apoptotic suggesting that B-1 cells are more resistant to apoptosis than B-2 cells. About 55% of macrophages showed apoptosis after infection (Figure 3C). Additionally, *in vitro* infection with A/WSN/1933 virus, but not UV-WSN virus, resulted in apoptotic death in the mouse macrophage cell line RAW 264.7 (Figure S6C). These results suggested that A/WSN/1933 virus induces apoptotic cell death specifically in the peritoneal cavity and RAW264.7 can be a model cell line to study this phenomenon. Considering these results together with Figure 1, peritoneal B cells survived after viral infection are major contributor to A/WSN/1933 virus-reactive antibody production in the peritoneal cavity of A/WSN/1933 virus-infected mice. To verify whether apoptosis was the plausible cause of peritoneal cell depletion, we carried out western blot analysis of proteins involved in apoptosis. The expression of cleaved PARP and cleaved Caspase-3 was increased by A/WSN/1933 virus infection in a time-dependent manner, while the expression of anti-apoptotic Bcl-2 was decreased (Figure 3D). These results demonstrated that live A/WSN/1933 virus induced apoptotic death of mouse peritoneal cells *in vitro*.

### Peritoneal Cells Possess Specific Receptors for Influenza A Virus

**Figure 4:**
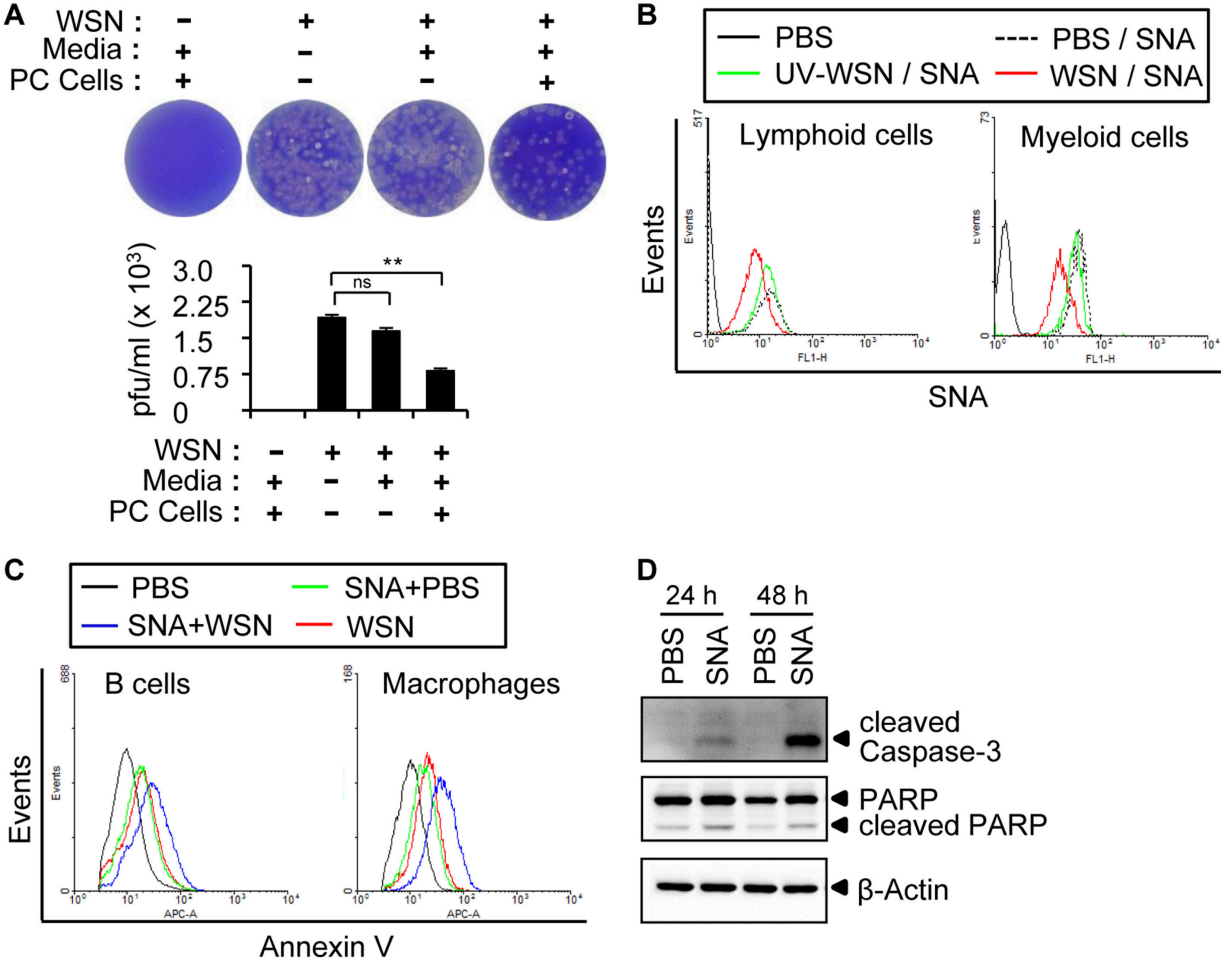
A/WSN/1933 virus binds to peritoneal cells. **(A)** Peritoneal cells were harvested from BALB/c mice (*n* = 3) and seeded into 5 ml round-bottom tubes (1 × 10^6^ cells in 1 ml). The tubes were treated with PBS or A/WSN/1933 virus (WSN, 3 × 10^3^ pfu). After 1 h of incubation at 4°C, the supernatant was separated by centrifugation and 100 μl of the supernatant was seeded along with MDCK cells in 6-well plates, incubated for 72 h, and stained with crystal violet, and plaques were counted. ns, not significant. ^**^P < 0.005. **(B)** Competitive binding of A/WSN/1933 virus and SNA to α-2,6-linked sialic acids of peritoneal cells. Peritoneal cells (1 × 10^6^) were incubated with 1 × 10^6^ pfu of A/WSN/1933 virus or UV-WSN virus for 1 h at 4°C and then treated with fluorescein-conjugated SNA for 1 h at 4°C. The cells were stained with fluorescence-conjugated antibodies (CD19 and B220 for lymphoid cells, CD11b for F4/80 myeloid cells) and then analyzed by flow cytometry. **(C)** Peritoneal cells were incubated in 24-well plates with SNA for 1 h at 37 °C and then treated with PBS (SNA+PBS), or 1 × 10^6^ pfu of A/WSN/1933 virus (SNA+WSN) for 72 h. Peritoneal cells treated with PBS only or A/WSN/1933 virus plus PBS were used as controls. The cells were stained with fluorescence-conjugated antibodies (CD19 and B220 for lymphoid cells, CD11b and F4/80 for myeloid cells) and Annexin V and then analyzed by flow cytometry. Triplicate experiments were carried out. **(D)** Expression of apoptotic proteins (cleaved Caspase-3 and cleaved PARP) in the PBS-treated control and SNA-treated peritoneal cells was determined by western blotting.

To investigate whether the A/WSN/1933 virus-induced apoptosis of peritoneal cells is caused by direct binding of the viruses, we decided to check the cellular receptors for influenza viruses. Following intranasal administration, influenza A virus initiates pathogenesis in the host by binding to specific sialic acid receptors on host cells (37). We first incubated A/WSN/1933 virus with media control or peritoneal cells for 1 h, and then the supernatants after centrifugation were used for infection of MDCK cells. Binding of the virus to the peritoneal cells would result in a reduction in viral particles in the supernatant and, therefore, a reduction in the subsequent MDCK infection as measured by plaque numbers. Indeed, infection of MDCK cells with peritoneal cell-treated viral supernatant resulted in significant reduction of plaque numbers compared to cells infected directly with virus. A slight decrease in plaque numbers was also observed upon incubation of the virus with culture medium alone; however, this was not statistically significant (Figure 4A). These results indicated that the A/WSN/1933 virus binds to peritoneal cells, at least transiently.

It is well-established that human influenza A virus binds to sialic acids linked to the penultimate galactose residue of cellular glycoproteins via α-2,6 glycosidic bonds (26). The presence of α-2,3-Gal and α-2,6-Gal sialic acid residues in the peritoneal cavity B cells of BALB/c mice has been reported (38). However, the role of peritoneal cavity-based sialic acids in relation to influenza virus infection has not been investigated. Using FITC-conjugated lectin staining with *Sambucus nigra* agglutinin (SNA) and *Maackia Amurensis* Lectin II (MAL II), which specifically recognize α-2,6-linked and α-2,3-linked sialic acids, respectively, we confirmed the occurrence of both α-2,6-linked and α-2,3-linked sialic acids in the lymphocytes and macrophages of the peritoneal cavity (Figures S7A,B) and spleen (Figures S7C,D) by flow cytometry. To determine whether A/WSN/1933 virus was able to bind to these sialic acids, we pre-incubated peritoneal cells with A/WSN/1933 virus or UV-WSN virus before treatment with FITC-conjugated SNA. We observed diminished binding of SNA to peritoneal cells in the presence of virus (Figure 4B), with UV-WSN virus inhibiting SNA binding much less efficiently than A/WSN/1933 virus, suggesting structural alterations during UV irradiation. These findings confirmed that A/WSN/1933 virus binds to α-2,6 sialic acids present on the cells of the peritoneal cavity.

Next, to determine whether SNA-induced blocking of A/WSN/1933 virus binding to cells reduces cell death, we pre-incubated peritoneal cells with SNA and then infected them with A/WSN/1933 virus. Surprisingly, enumeration of Annexin V-stained cells revealed that SNA itself induced apoptosis of both B cells and macrophages (Figure 4C), and infection with the virus compounded the effect (Figure 4C). The expression of cleaved PARP and caspase-3 in peritoneal cells was increased by SNA treatment (Figure 4D). SNA-induced apoptosis was also observed in the mouse macrophage cell line RAW 264.7 (Figure S8A). However, *in vitro* infection of spleen B cells and T cells with A/WSN/1933 virus or treatment of these cells with SNA did not induce any significant changes in the numbers of Annexin V-stained cells (Figure S8B). These results demonstrated that the interaction of α-2,6 sialic acids with SNA and/or A/WSN/1933 virus specifically promoted apoptosis of peritoneal cells and RAW 264.7 cells.

### Repeated Infection Boosts Antibody Production With Limited B Cell Depletion

**Figure 5:**
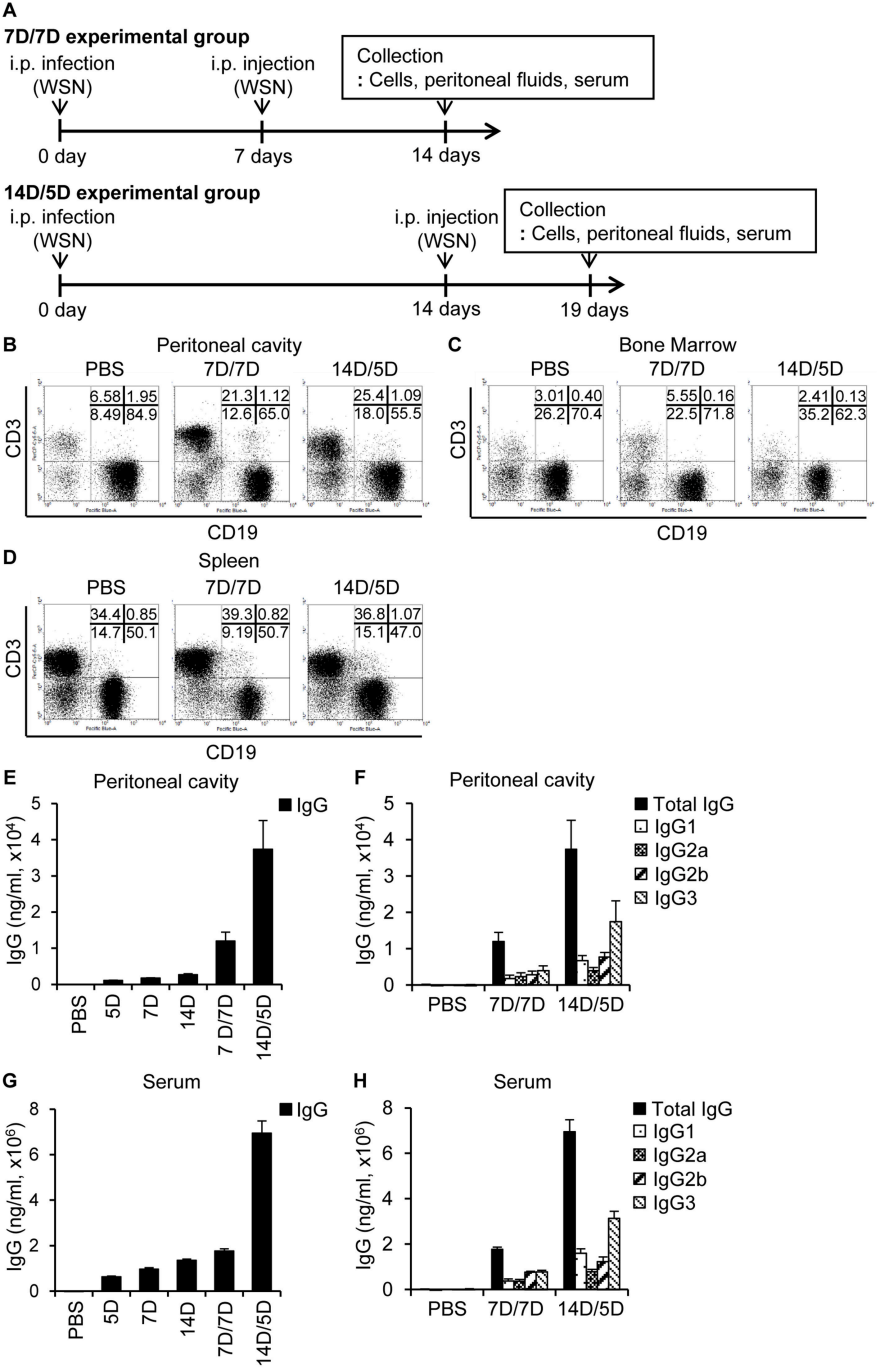
Re-infection with A/WSN/1933 virus limits B cell death and induces antibody production. **(A)** Schematic diagram of the experimental procedure. BALB/c mice (*n* = 5/group) were sacrificed after repeated intraperitoneal inoculation with PBS or 5 × 10^6^ pfu of A/WSN/1933 virus. 7D/7D represents a procedure consisting of a first infection, a second infection after 7 days, and then sacrifice after another 7 days. 14D/5D represents a procedure of first infection, second infection after 14 days, and then sacrifice after another 5 days. Peritoneal cavity cells **(B)**, bone marrow cells **(C)**, and splenocytes **(D)** (FSC^low^SSC^low^) were gated, and stained with fluorescence-conjugated antibodies (CD19, B220) and then analyzed by flow cytometry. **(E–H)** Peritoneal cavity fluids and sera were collected from the mice and the amounts of total IgG **(E,G)** and IgG subclasses **(F,H)** in the peritoneal cavity **(E,F)** and serum **(G,H)** were quantified by ELISA. Figures are representative of three replicate experiments.

Following primary intraperitoneal inoculation of A/WSN/1933 virus, we observed a marked reduction of peritoneal and bone marrow B cells 5 days post-infection, with gradual recovery to normal levels 14 days post-infection (Figure 2). To examine the effect of repeated infection on B cells *in vivo*, we reinoculated BALB/c mice with A/WSN/1933 virus using two schedules; reinoculation 7 days after the first infection followed by mice sacrifice 7 days later (7D/7D) and reinoculation 14 days after the first infection followed by mice sacrifice 5 days later (14D/5D). In both schedules, flow cytometric analysis (FSC^low^SSC^low^) showed a significant increase in T cells (CD3 positive populations: from 6.58% to 21.3% and 25.4%) but comparatively mild decrease in B cells (CD19 positive populations: (from 84.9% to 65.0% and 55.5%) in the peritoneal cavity (Figure 5A). Total B cell number was recovered to normal level in the experimental group 14D/5D (Figure S9A). In bone marrow, the population of T cells was increased in the experimental group 7D/7D (3.01% to 5.55%) but there was no prominent change in B cells (Figure 5B). Total B cell number was increased in the experimental group 7D/7D (Figure S9B). In spleen, the cell population was almost similar to those of the uninfected control mice (Figure 5C, Figure S9C). These results indicate that the second intraperitoneal inoculation does not induce cell depletion to the same extent as the first inoculation. This protection from the second inoculation might be provided by the neutralizing antibodies produced in the peritoneal cavity during the primary exposure to the virus. A/WSN/1933 virus-reactive total IgG and subclasses (IgG1, IgG2a, IgG2b, and IgG3) in both the peritoneal cavity (Figures 5D,E) and the serum (Figures 5F,G) were significantly higher in the 14D/5D set than in the 7D/7D set, with IgG3 being the major subclass produced. In conclusion, repeated intraperitoneal infection of mice with the same virus induced significantly increased amounts of virus-reactive IgG in the peritoneal cavity as well as in the serum without drastic change of B cell population. This protection from the second inoculation might be provided by the neutralizing antibodies produced in the peritoneal cavity during the primary exposure to the virus.

### A/WSN/1933 Virus-Reactive Antibodies Exhibit Cross-Reactivity Against Other Influenza A Viruses

**Figure 6:**
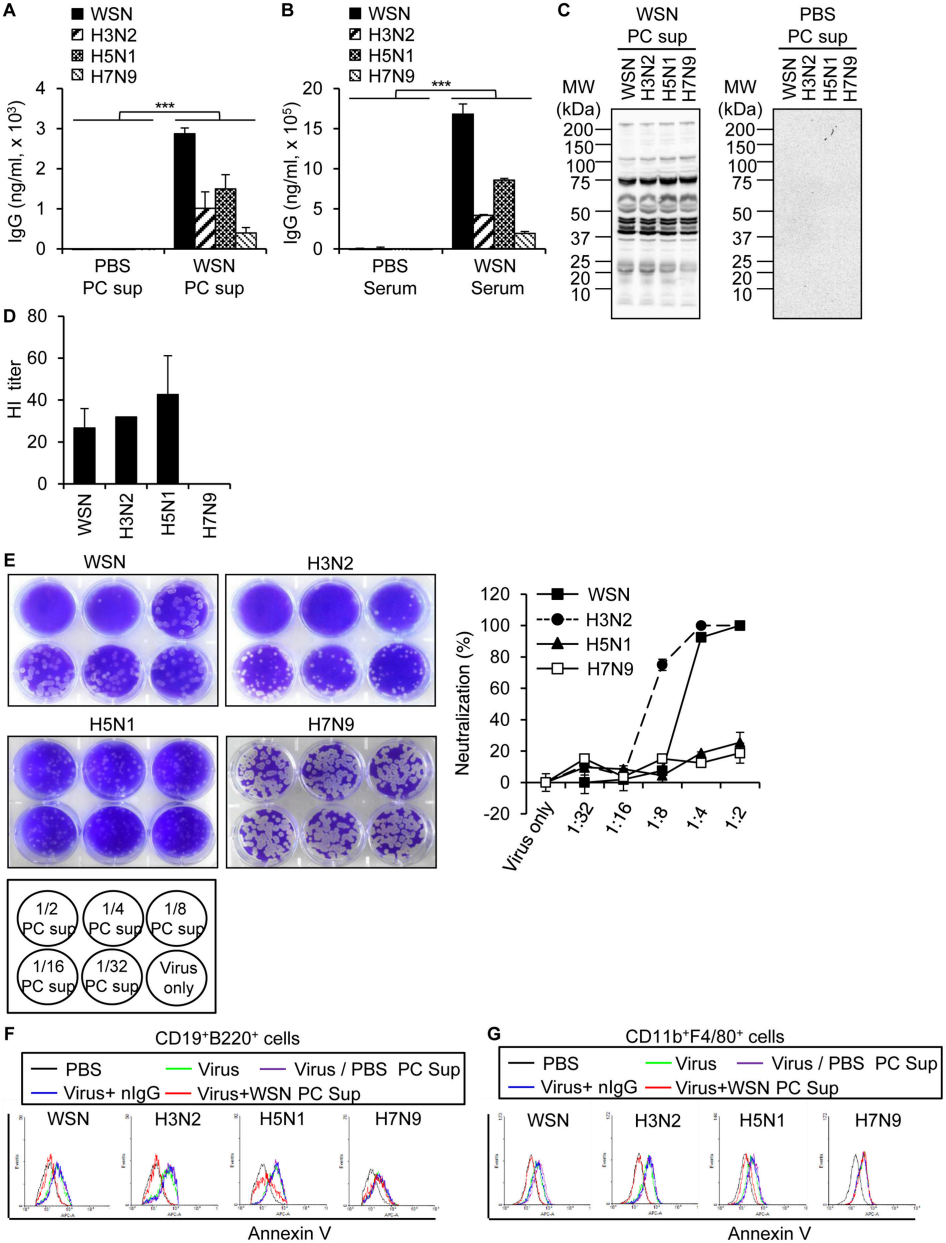
Cross reactivity of A/WSN/1933 virus-induced antibody to other influenza A viruses. Peritoneal cavity fluids and sera were collected from BALB/c mice (*n* = 5/group) 14 days after intraperitoneal inoculation with 5 × 10^6^ pfu of A/WSN/1933 virus. **(A,B)** Amounts of IgG cross-reactive against other influenza A viruses in peritoneal cavity fluids **(A)** and sera **(B)** obtained from A/WSN/1933 virus-inoculated mice were quantified by ELISA. ^***^p < 0.0005. **(C)** Identification of cross-reactivity by western blotting. Influenza protein samples from influenza A viruses were separated by SDS-PAGE and then examined by western blot analysis with the peritoneal cavity fluids of A/WSN/1933 virus-inoculated (WSN PC sup) or PBS-inoculated (PBS PC sup) mice. **(D)** Identification of cross reactivity by a hemagglutination inhibition (HI) assay. HI titers of the peritoneal cavity fluids from A/WSN/1933 virus-injected BALB/c were performed with 4 hemagglutination units (4HA) of each influenza A virus. **(E)** Identification of cross-reactivity by virus neutralization assay. The ability of the peritoneal cavity fluids of A/WSN/1933 virus-inoculated mice to neutralize the plaque forming ability of each influenza A virus was determined by counting plaques and calculating the neutralization percentage. **(F,G)** PBS-injected peritoneal cavity fluids (PBS PC Sup), A/WSN/1933 virus-inoculated peritoneal cavity fluids (WSN PC Sup), or mouse normal IgG (nIgG) were incubated with the indicated influenza A viruses for 1 h and then incubated with peritoneal cells. **(F)** After 72 h of incubation, the cells were stained with fluorescence-conjugated antibodies (CD19, B220) and Annexin V and then analyzed by flow cytometry. Annexin V-positive B cells are represented in histograms. **(G)** After 72 h of incubation, the cells were stained with fluorescence-conjugated antibodies (CD11b, F4/80) and Annexin V and then analyzed by flow cytometry. Annexin V-positive macrophages are represented in histograms. PC Sup, Peritoneal cavity fluids. WSN, A/WSN/1933 (H1N1). H3N2, A/Hongkong/4801/2014 (H3N2). H5N1, rIETR CVV (H5N1). H7N9, NIBRG-268M (H7N9).

In order to examine the degree of protection conferred by A/WSN/1933 virus-induced antibodies in the peritoneal cavity, we analyzed the reactivity of peritoneal cavity fluid to A/WSN/1933 virus as well as its cross-reactivity to three other influenza A viruses: wt A/Hongkong/4801/2014 (H3N2), rIETR CVV (H5N1), NIBRG-268M (H7N9). ELISA revealed that A/WSN/1933 virus-reactive IgG in the peritoneal cavity fluids (Figure 6A) and serum (Figure 6B) obtained 14 days after infection bound to A/WSN/1933 virus with the highest efficiency, and with less efficiency to the other influenza viruses. In addition, western blot analysis revealed that the peritoneal cavity fluids of A/WSN/1933 virus-infected mice recognized multiple protein bands presumably corresponding to the influenza proteins of all viruses under study (Figure 6C). In contrast, the peritoneal cavity fluids from the PBS-injected mice didn't recognize the protein bands. Furthermore, the peritoneal cavity fluids of A/WSN/1933 virus-infected mice could inhibit hemagglutination mediated by all influenza A viruses, except NIBRG-268M (H7N9) virus (Figure 6D). However, the peritoneal cavity fluids neutralized the plaque-forming ability of A/WSN/1933 virus and A/Hongkong/4801/2014 (H3N2) virus, but not that of rIETR CVV (H5N1) virus and NIBRG-268M (H7N9) virus on MDCK cells (Figure 6E).

We hypothesized that peritoneal cavity fluids from A/WSN/1933 virus-infected mice could neutralize apoptosis, therefore we determined the effect of peritoneal cavity fluids from A/WSN/1933 virus-infected mice on apoptosis induced by A/WSN/1933 virus and the other influenza A viruses. For this purpose, peritoneal cells that were pre-incubated with peritoneal cavity fluids from A/WSN/1933 virus-infected mice were incubated with each influenza A virus. Flow cytometric analysis of Annexin V stained-peritoneal cells revealed neutralization of A/WSN/1933 virus-, rIETR CVV (H5N1) virus- and A/Hongkong/4801/2014 (H3N2) virus-, but not NIBRG-268M (H7N9) virus-induced apoptosis of B cells (Figure 6F) and macrophages (Figure 6G), which is likely to be mediated by virus-reactive antibodies in the peritoneal cavity fluid. In contrast, preincubation of each virus with peritoneal cavity fluids from PBS-injected mice or normal mouse IgG (nIgG) had no effect on virus-induced apoptosis (Figures 6F,G). Our results suggest that while the antibody against one influenza A virus can broadly bind to other influenza strains, its functional responses, such as hemagglutination inhibition, plaque neutralization and apoptosis inhibition, may be restricted to fewer viruses.

### A/WSN/1933 Virus Infection Induces Cross-Protection Against A/Hongkong/4801/2014 (H3N2) Virus

**Figure 7:**
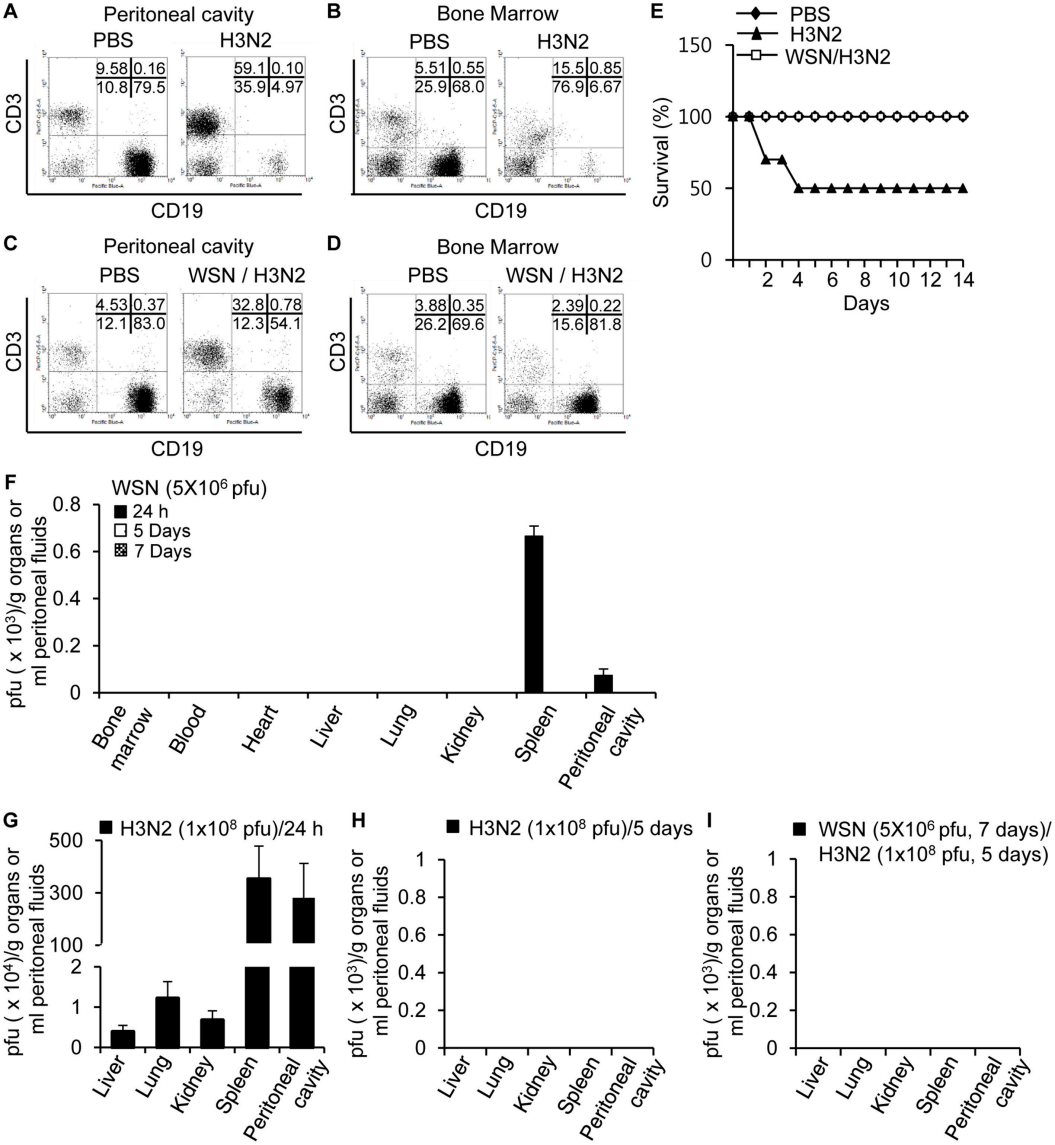
Cross-protection of A/WSN/1933 virus inoculation against lethal dose of A/Hongkong/4801/2014 (H3N2) virus. **(A,B)** BALB/c mice (*n* = 5) were sacrificed 5 days after a single intraperitoneal challenge with 1 × 10^8^ pfu of wt A/Hong Kong/4801/2014 (H3N2) virus, and the populations of peritoneal cavity B cells **(A)** and bone marrow B cells **(B)** were analyzed by flow cytometry. **(C,D)** BALB/c mice (*n* = 10) were first inoculated with A/WSN/1933 virus (5 × 10^6^ pfu) and then inoculated with A/Hongkong/4801/2014 (H3N2) (1 × 10^8^ pfu) after 7 days, followed by sacrifice 5 days after the second challenge. The B cell populations of the peritoneal cavity **(C)** and bone marrow **(D)** were characterized by flow cytometry. **(E)** BALB/c mice (*n* = 10) were first inoculated with A/WSN/1933 virus (5 x 10^6^ pfu), and a second inoculation with A/Hongkong/4801/2014 (H3N2) (1 × 10^8^ pfu) was performed after 7 days. The mice were monitored for 14 days, and survival percentages of the mice were measured. **(F)** BALB/c mice were infected intraperitoneally with 5 × 10^6^ pfu of A/WSN/1933 virus (*n* = 5/group). After 24 h, 5 days and 14 days, A/WSN/1933 virus titers in the indicated organs (pfu/g), blood and peritoneal fluids (pfu/ml) were determined by plaque assay. **(G,H)** BALB/c mice were infected intraperitoneally with 1 x 10^8^ pfu of A/Hongkong/4801/2014 (H3N2) virus (*n* = 15/group). After 24 h (*n* = 5), virus titers in the indicated organs (pfu/g) and peritoneal fluids (pfu/ml) were determined by plaque assay **(G)**. After 5 days, virus titers in the indicated organs (pfu/g) and peritoneal fluids (pfu/ml) of A/Hongkong/4801/2014 (H3N2) virus-infected mice (live mice, *n* = 5) were determined by plaque assay **(H)**. **(I)** BALB/c mice (*n* = 5) were first intraperitoneally inoculated with A/WSN/1933 virus (5 × 10^6^ pfu), and a second inoculation with A/Hongkong/4801/2014 (H3N2) (1 × 10^8^ pfu) was performed after 7 days. After another 5 days, virus titers in the indicated organs (pfu/g) and peritoneal fluids (pfu/ml) were determined by plaque assay. WSN, A/WSN/1933 virus. H3N2, A/Hongkong/4801/2014 (H3N2) virus.

Since peritoneal cavity fluids obtained from A/WSN/1933 virus-infected mice inhibited hemagglutination (Figure 6D), plaque formation (Figure 6E), and apoptosis (Figures 6F,G) mediated by the A/Hongkong/4801/2014 (H3N2) strain, we decided to investigate the protective effect of A/WSN/1933 virus inoculation on A/Hongkong/4801/2014 (H3N2) virus infection *in vivo*. For this purpose, we first injected BALB/c mice intraperitoneally with different doses of H3N2 virus to determine the optimal dose for the infection following A/WSN/1933 virus inoculation. We observed 50% mortality 5 days after challenge with A/Hongkong/4801/2014 (H3N2) at the highest dose tested (1 × 10^8^ pfu), whereas infection with lower doses resulted in 100% survival (Figure S10). As with A/WSN/1933 virus infection (Figures 2A,B), massive loss of peritoneal cavity B cells (Figure 7A) and bone marrow B cells (Figure 7B) was observed at day 5 post-infection in mice that survived the high-dose A/Hongkong/4801/2014 (H3N2) virus infection. Marked infiltration of T cells in the peritoneal cavity was also found (Figure 7A). Next, to examine the protective effect of A/WSN/1933 virus infection, BALB/c mice intraperitoneally infected with A/WSN/1933 virus were intraperitoneally inoculated with a high dose of A/Hongkong/4801/2014 (H3N2) virus 7 days later and sacrificed after 5 days to test for any changes in B cell populations in the peritoneal cavity and bone marrow. Subsequent exposure to A/Hongkong/4801/2014 (H3N2) virus had comparatively mild or minor effect on B cell populations in the peritoneal cavity (from 83.0 to 54.1%, Figure 7C) or the bone marrow (69.6 to 81.8%, Figure 7D). We found that while mice infected only with A/Hongkong/4801/2014 (H3N2) virus exhibited only 50% survival within 5 days, the mice initially inoculated with A/WSN/1933 virus and then exposed to the A/Hongkong/4801/2014 (H3N2) virus exhibited 100% survival for up to 14 days after A/Hongkong/4801/2014 (H3N2) virus infection (Figure 7E). These results suggest that A/WSN/1933 virus infection can protect against future infection with a fatal dose of the A/Hongkong/4801/2014 (H3N2) virus.

We examined the presence of A/WSN/1933 virus following intraperitoneal inoculation. There was no sign of replication of influenza virus in the peritoneal cells. In early time points after virus inoculation (5 × 10^6^ pfu/mouse, 24 h), the presence of A/WSN/1933 virus was confirmed by plaque assays in the spleen and peritoneal cavity. However, we could not detect the virus in any of these organs from infected mice when we examined at 5 to 14 days post-infection (Figure 7F). Furthermore, we tried virus infection *in vitro* in cells of the peritoneal cavity. However, we could not detect the virus amplification. Next, we measured virus titers in the peritoneal cavity, liver, lung, kidney, and spleen after intraperitoneal infection of H3N2 (1 × 10^8^ pfu/mouse). After 24 h, the viruses were found in all the examined organs (Figure 7G). However, we could not detect viruses anywhere 5 days after infection (Figure 7H). Therefore, it is likely that H3N2 influenza viruses don't replicate in mice, either. When the mice were infected first with A/WSN/1933 virus and then superinfected with H3N2 later, there were no viruses found in the examined organs (Figure 7I). Considering these data, we speculate that the A/WSN/1933 virus infection induces immune responses which can protect mice against A/Hongkong/4801/2014 (H3N2) virus infection independently of virus replication.

## Discussion

In development of vaccines against viral diseases, the route of infection or vaccination is an important issue because there can be immunological differences depending on the routes (4–9). Previously, other investigators showed that intraperitoneal inoculation of live influenza A virus induced protection against intranasal infections in mouse and ferret models (16–19). Here, we investigated immune responses in the mouse peritoneal cavity after intraperitoneal infection with influenza A virus in detail and found a possibility that the immune responses in the peritoneal cavity is unique and effective in terms of antibody production and its cross-reactivity. As we are focusing on immune responses in the peritoneal cavity here, we used intraperitoneal infection rather than intranasal infection in this study to assess protection conferred by intraperitoneal immunization.

It was previously reported that live influenza viruses are more powerful than inactivated vaccines when administered intramuscularly or intranasally (8, 36). Here, we compared the immunogenicity of live and inactivated A/WSN/1933 virus after intraperitoneal inoculation and found that the amounts of virus-reactive antibodies at various time points remained significantly higher (approximately 6~10 fold) in the live A/WSN/1933 virus-infected mice than in the mice infected with UV-inactivated A/WSN/1933 virus, supporting the superior effect of the live virus.

Since the influenza A virus has been detected in the lungs, heart, blood, kidney, brain, spleen and bone marrow by hemagglutination assays and plaque assays after intranasal inoculation (9), we examined the presence of A/WSN/1933 virus following intraperitoneal inoculation in these tissues along with peritoneal cells and fluids. There was no sign of replication of influenza virus in the peritoneal cells. In early time points after virus inoculation, the presence of A/WSN/1933 virus was confirmed by plaque assays in in the lungs, heart, blood, kidney, spleen and bone marrow. However, we could not detect the virus in any of these organs from infected mice when we examined at 5 to 14 days post-infection. These results suggest that immune responses to influenza A virus in the peritoneal cavity result in virus-reactive antibody production and these antibodies efficiently block the viruses in peritoneal cavity as well as in other organs.

B-1 cells produce natural IgM antibody against infection in the infected region. In the respiratory tract influenza virus infection, it was reported that B-1 cells produce natural IgM antibody in the airway space (39, 40). In contrast, there is no report regarding production of natural influenza-binding IgM by B-1 cells in response to intramuscular infection of influenza A virus till now. In this study, we also found increased production of A/WSN/1933-binding IgM in response to inoculation of A/WSN/1933 virus in the peritoneal cavity at early time points. CD4^+^ T cell-independent B cell response might occur frequently in case of peritoneal infection because B-1 cells are dominant population in the peritoneal cavity. Therefore, it can be an advantageous factor that can explain the potent activity of peritoneal cells. As we didn't directly compare immune responses of intraperitoneal infection with those of intranasal or intramuscular infection, we can't define the importance of B-1 cells population ratio at this time.

Intranasal infection of mice with influenza A virus induces severe loss of blood lymphocytes, bone marrow cells and lung B cells resulting in malfunction of the immune system (20–23). Apoptosis has been attributed as the cause of bone marrow and spleen B cell death following virus infections (20–24). Influenza A virus has also been reported to induce apoptosis of human airway epithelial cells (41). Consistent with these reports, we show that intraperitoneal infection with A/WSN/1933 virus resulted in massive but transient peritoneal and bone marrow B cell depletion. *In vitro* infection of peritoneal cells with A/WSN/1933 virus increased the populations of Annexin V-stained B cells and macrophages. Furthermore, apoptotic death of peritoneal cells was associated with increased expression of leaved PARP and Caspase-3 along with reduced levels of Bcl-2. This is consistent with previously reported inhibition of influenza A virus-induced apoptosis in MDCK cells by Bcl-2 (42). The mouse macrophage cell line RAW 264.7, that can be infected by influenza A virus (43), also underwent apoptosis upon infection with A/WSN/1933 A virus. These results suggest that apoptosis is responsible for the depletion of B cells and macrophages in the peritoneal cavity. Importantly, the peritoneal cavity including the antiviral IgG inhibited the influenza A virus-induced apoptosis of peritoneal B cells and macrophages.

Even though the peritoneal cavity cells were subjected to apoptosis, production of A/WSN/1933 virus-reactive IgM and IgG decreased and increased over time, respectively, suggesting class switching of antibody producing B cells. As B-1 cells are more resistant to apoptosis induced by A/WSN/1933 virus infection than B-2 cells, B-1 cells may be involved in the CD4^+^ T cell-independent antibody response at early stage and CD4^+^ T cell-dependent antibody responses may occur later. When we analyzed populations of B cells in the peritoneal cavity at 14 days post-infection, we observed increase in the numbers of B cells and B-1 cells compared to control mice. We also observed increase of IgG^+^ B-1 and IgG^+^ B-2 cells at 14 days post-infection. From our results, major key population producing virus-reactive antibodies is still unclear. Therefore, the contribution of B-1 and B-2 cells to production of A/WSN/1933-reactive antibodies is to be determined.

A prior study revealed the accumulation of macrophages and neutrophils in the lung soon after intranasal inoculation with A/WSN/1933 virus (44). An increase in neutrophil infiltration and its protective role during influenza A virus infection following intranasal infection has been described (45). We found that intraperitoneal challenge with A/WSN/1933 virus led to marked depletion of macrophages from the peritoneal cavity at 12 h post-inoculation, while immense neutrophil infiltration occurred. These findings suggest that neutrophils may be important in the immunological responses and protection that follow intraperitoneal challenge with A/WSN/1933 virus.

Human influenza virus binds to α-2,6-linked sialic acids and avian influenza virus to α-2,3-linked sialic acids (46). Macrophage galactose-type receptors and mannose receptors are also involved in the binding of influenza A virus (43). Recently, the sialic acid receptors have been detected in the peritoneal cavity cells of mice (38). Here, we demonstrated the interaction between peritoneal cells and influenza A virus and confirmed the occurrence of both α-2,6-linked and α-2,3-linked sialic acids in B cells and macrophages of the peritoneal cavity. The existence of dual α-2,6-linked and α-2,3-linked sialic acids has been reported previously in alveolar macrophages (47). We suggest that following intraperitoneal infection, A/WSN/1933 virus binds to α-2,6-linked sialic acids and then induces apoptotic death of macrophages and B cells in the peritoneal cavity. Considering that splenic B cells also express α-2,6 sialic acid but apoptosis of splenic B cells was not induced by A/WSN/1933, it is likely that there can be some differential signaling after A/WSN/1933 binding. The difference between the splenic B cells and peritoneal cells has to be investigated in future work.

Next, we noticed that repeated infection with A/WSN/1933 virus did not have adverse effects on B cells or macrophages; we attributed this to the neutralizing antibodies produced in response to the first exposure. We also perceived a significant increase in the A/WSN/1933 virus-reactive IgG production following repeated infection with A/WSN/1933 virus. The proliferation of A/WSN/1933 virus-reactive B cells may be responsible for the higher antibody response and therefore increased protection after re-infection. Conversely, repeated intranasal infection with low-dose influenza virus results in severe disease in mice (48). These results suggest that the immune responses against influenza A virus operate differently in the peritoneal cavity and the lung airway. We speculate that while extensive depletion of immune cells occurs in response to virus infection, A/WSN/1933 virus-reactive immune cell populations are selected and expanded in the peritoneal cavity.

It has been reported that cross-protection against pandemic H1N1 virus can be induced by seasonal H1N1 virus infection via a CD8^+^ T cell-independent, B cell-dependent mechanism (49). Similarly, the intranasal administration of gamma-irradiated A/PR8/H1N1 virus preparation was protective against seasonal and avian H5N1 influenza virus infections (50). Here, we found that the antibodies produced by challenge with intraperitoneal inoculation with A/WSN/1933 virus were poly-reactive to various influenza viruses, such as A/Hongkong/4801/2014 (H3N2) virus, rIETR CVV (H5N1) virus, and NIBRG-268M (H7N9) virus, explaining the cross-reactive protection by the influenza vaccines. However, our further finding that peritoneal cavity fluid inhibited hemagglutination of only A/Hongkong/4801/2014 (H3N2) virus and rIETR CVV (H5N1) virus and plaque formation by only A/Hongkong/4801/2014 (H3N2) virus may account for the limited function of influenza vaccines in terms of cross-reactivity. This phenomenon can be understood in the context that influenza viruses have different antigenic sites and glycosylation sites in the neighboring region of receptor binding domains and different amino acid sequences in the receptor binding domains of HA. Functional inhibition of the antibody against influenza infection may need more specific blocking of the receptor binding domain and neighboring region of receptor binding domains of the viruses than simple binding reaction. Even though the antibody cannot neutralize rIETR CVV (H5N1) virus in MDCK cells, the antibody protects apoptosis of B cells in response to rIETR CVV (H5N1) virus. It suggests that blocking of infection needs more accurate or potent structural complementarity than blocking of apoptosis.

Previously, H3N2 was reported not to cause severe disease or death in BALB/c mice with dose of 10^5^ pfu (51–54). Interestingly, we found that intraperitoneal infection at a high dose of A/Hongkong/4801/2014 (H3N2) virus (1 × 10^8^ pfu) induced the death of BALB/c mice without sign of virus replication. We expect that this phenomenon may have resulted from severe microvascular endothelial leakage of organs associated within the peritoneal cavity as previously reported (55, 56). It is also possible that there are other reasons unidentified. Therefore, further investigation of the detailed mechanism involved in the death of mice is necessary.

We observed that A/WSN/1933 virus infection induced immunity against lethal intraperitoneal A/Hongkong/4801/2014 (H3N2) virus exposure in BALB/c mice. This protection might be provided by A/WSN/1933 virus-reactive IgG in the peritoneal cavity, as explained by our *in vitro* assays. Given the role of increased magnitude of CD8^+^ T cell memory in response to sequential priming or reinfection (57, 58) and our results regarding the increased T cell population after A/WSN/1933 virus infection, repeated A/WSN/1933 virus infection may further enhance generation of immunological memory in our system. Therefore, this issue must be pursued in future studies to better understand the effect of vaccination.

In short, this is the first report showing the responses of peritoneal cells to intraperitoneal inoculation of influenza A virus. Our results suggest that the immunological responses in the peritoneal cavity can be greatly effective in response to virus infection in mice. It is unclear how we can apply this information to human system. However, we can postulate a possibility that there can be a route of choice for vaccination against influenza virus in humans; therefore, further studies on the effects of different vaccination routes are required. We have also shown that reinfection can induce increasingly powerful and cross-protective immune responses possibly through influenza A virus-induced antibody production. Considering that the influenza viruses undergo reassortment and evolve pathogenically, our results may provide a strategy for efficacious vaccine development against the continuous threat of new influenza A viruses.

## Ethics Statement

All animal procedures performed in this study are in accordance with the recommendations in the Guide for the Care and Use of Laboratory Animals of the National Veterinary Research & Quarantine Service of Korea. This study was approved by the Institutional Animal Care and Use Committee of Hallym University (Permit Number: Hallym2015-54 and 2017-41).

## Author Contributions

H-JK conceived of the project. H-JK, AG, M-SP, and YL designed the experiments and wrote the manuscript. AG, BP, TK, MA, DK, SM, JP, JK, and HL carried out the experiments; H-JK, AG, M-SP, and YL analyzed data. All authors approved the final version of the manuscript.

### Conflict of Interest Statement

The authors declare that the research was conducted in the absence of any commercial or financial relationships that could be construed as a potential conflict of interest.

## References

[1] Manicassamy B Medina RA Hai R Tsibane T Stertz S Nistal-Villan E. Protection of mice against lethal challenge with 2009 H1N1 influenza A virus by 1918-like and classical swine H1N1 based vaccines. PLoS Pathog. (2010) 6:e1000745. 10.1371/journal.ppat.1000745 20126449 PMC2813279

[2] Horimoto T Kawaoka Y. Influenza: lessons from past pandemics, warnings from current incidents. Nat Rev Microbiol. (2005) 3:591–600. 10.1038/nrmicro1208 16064053

[3] Mathews JD Chesson JM McCaw JM McVernon J. Understanding influenza transmission, immunity and pandemic threats. Influenza Other Respir Viruses. (2009) 3:143–49. 10.1111/j.1750-2659.2009.00089.x 19627371 PMC4634682

[4] Hauge S Madhun A Cox RJ Haaheim LR. Quality and kinetics of the antibody response in mice after three different low-dose influenza virus vaccination strategies. Clin Vaccine Immunol. (2007) 14:978–83. 10.1128/cvi.00033-07 17596426 PMC2044485

[5] Magiri R Lai K Chaffey A Zhou Y Pyo HM Gerdts V. Intradermal immunization with inactivated swine influenza virus and adjuvant polydi(sodium carboxylatoethylphenoxy)phosphazene (PCEP) induced humoral and cell-mediated immunity and reduced lung viral titres in pigs. Vaccine. (2018) 36:1606–13. 10.1016/j.vaccine.2018.02.026 29454517

[6] Diebold SS. Determination of T-cell fate by dendritic cells. Immunol Cell Biol. (2008) 86:389–97. 10.1038/icb.2008.26 18382438

[7] Durando P Iudici R Alicino C Alberti M de Florentis D Ansaldi F. Adjuvants and alternative routes of administration towards the development of the ideal influenza vaccine. Hum Vaccin. (2011) 7 (Suppl):29–40. 10.4161/hv.7.0.14560 21245655

[8] Harris K Ream R Gao J Eichelberger MC. Intramuscular immunization of mice with live influenza virus is more immunogenic and offers greater protection than immunization with inactivated virus. Virol J. (2011) 8:251. 10.1186/1743-422x-8-251 21600020 PMC3123286

[9] Nishimura H Itamura S Iwasaki T Kurata T Tashiro M. Characterization of human influenza A (H5N1) virus infection in mice: neuro-, pneumo- and adipotropic infection. J Gen Virol. (2000) 81:2503–10. 10.1099/0022-1317-81-10-2503 10993940

[10] Bodewes R Kreijtz JH van Amerongen G Hillaire ML Vogelzang-van Trierum SE Nieuwkoop NJ Infection of the upper respiratory tract with seasonal influenza A(H3N2) virus induces protective immunity in ferrets against infection with A(H1N1)pdm09 virus after intranasal, but not intratracheal, inoculation. J Virol. (2013) 87:4293–301. 10.1128/jvi.02536-12 23365444 PMC3624397

[11] Maroof A Yorgensen YM Li Y Evans JT. Intranasal vaccination promotes detrimental Th17-mediated immunity against influenza infection. PLoS Pathog. (2014) 10:e1003875. 10.1371/journal.ppat.1003875 24465206 PMC3900655

[12] Wang C Zhu W Luo Y Wang BZ. Gold nanoparticles conjugating recombinant influenza hemagglutinin trimers and flagellin enhanced mucosal cellular immunity. Nanomedicine. (2018) 14:1349–60. 10.1016/j.nano.2018.03.007 29649593 PMC6177327

[13] Knuschke T Sokolova V Rotan O Wadwa M Tenbusch M Hansen W. Immunization with biodegradable nanoparticles efficiently induces cellular immunity and protects against influenza virus infection. J Immunol. (2013) 190:6221–29. 10.4049/jimmunol.1202654 23667109

[14] Reading PC Whitney PG Pickett DL Tate MD Brooks AG. Influenza viruses differ in ability to infect macrophages and to induce a local inflammatory response following intraperitoneal injection of mice. Immunol Cell Biol. (2010) 88:641–50. 10.1038/icb.2010.11 20142836

[15] Deng L Ibanez LI Van den Bossche V Roose K Youssef SA de Bruin A. Protection against influenza A virus challenge with M2e-displaying filamentous *Escherichia coli* Phages. PLoS ONE. (2015) 10:e0126650. 10.1371/journal.pone.0126650 25973787 PMC4431709

[16] Francis T Magill TP. Quantitative relationships between the immunizing dose of epidemic influenza virus and the resultant immunity. J Exp Med. (1939) 69:283–300. 10.1084/jem.69.2.283 19870847 PMC2133739

[17] To KK Zhang AJ Chan AS Li C Cai JP Lau CC. Recombinant influenza A virus hemagglutinin HA2 subunit protects mice against influenza A(H7N9) virus infection. Arch Virol. (2015) 160:777–86. 10.1007/s00705-014-2314-x 25616843

[18] Razin MAF Osman A Ali MA Bahgat MM Maghraby AS. Immune responses to killed reassorted influenza virus supplemented with natural adjuvants. Acta Microbiol Immunol Hung. (2017) 64:313–30. 10.1556/030.64.2017.011 28627238

[19] Xu W Zheng M Zhou F Chen Z Long-term immunogenicity of an inactivated split-virion 2009 pandemic influenza A H1N1 virus vaccine with or without aluminum adjuvant in mice. Clin Vaccine Immunol. (2015) 22:327–335. 10.1128/cvi.00662-14 25589552 PMC4340896

[20] Sedger LM Hou S Osvath SR Glaccum MB Peschon JJ van Rooijen N. Bone marrow B cell apoptosis during *in vivo* influenza virus infection requires TNF-alpha and lymphotoxin-alpha. J Immunol. (2002) 169:6193–201. 10.4049/jimmunol.169.11.6193 12444124

[21] Borrow P Hou S Gloster S Ashton M Hyland L. Virus infection-associated bone marrow B cell depletion and impairment of humoral immunity to heterologous infection mediated by TNF-alpha/LTalpha. Eur J Immunol. (2005) 35:524–532. 10.1002/eji.200425597 15657949

[22] Tumpey TM Lu X Morken T Zaki SR Katz JM. Depletion of lymphocytes and diminished cytokine production in mice infected with a highly virulent influenza A (H5N1) virus isolated from humans. J Virol. (2000) 74:6105–6116. 10.1128/jvi.74.13.6105-6116.2000 10846094 PMC112109

[23] Dougan SK Ashour J Karssemeijer RA Popp MW Avalos AM Barisa M. Antigen-specific B-cell receptor sensitizes B cells to infection by influenza virus. Nature. (2013) 503:406–9. 10.1038/nature12637 24141948 PMC3863936

[24] Qi W Tian J Zhang C He J Ning Z Jiao P. Potential role of HPA axis and sympathetic nervous responses in depletion of B cells induced by H9N2 avian influenza virus infection. PLoS ONE. (2012) 7:e51029. 10.1371/journal.pone.0051029 23251416 PMC3519482

[25] Bouvier NM Lowen AC. Animal models for influenza virus pathogenesis and transmission. Viruses. (2010) 2:1530–1563. 10.3390/v20801530 21442033 PMC3063653

[26] Qi L Kash JC Dugan VG Wang R Jin G Cunningham RE. Role of sialic acid binding specificity of the 1918 influenza virus hemagglutinin protein in virulence and pathogenesis for mice. J Virol. (2009) 83:3754–61. 10.1128/jvi.02596-08 19211766 PMC2663248

[27] Ning ZY Luo MY Qi WB Yu B Jiao PR Liao M. Detection of expression of influenza virus receptors in tissues of BALB/c mice by histochemistry. Vet Res Commun. (2009) 33:895–903. 10.1007/s11259-009-9307-3 19662506

[28] Lee I Il Kim J Park S Bae JY Yoo K Yun SH. Single PA mutation as a high yield determinant of avian influenza vaccines. Sci Rep. (2017) 7:40675. 10.1038/srep40675 28084423 PMC5233958

[29] Rhee JW Kim D Park BK Kwon S Cho S Lee I. Immunization with a hemagglutinin-derived synthetic peptide formulated with a CpG-DNA-liposome complex induced protection against lethal influenza virus infection in mice. PLoS ONE. (2012) 7:e48750. 10.1371/journal.pone.0048750 23144954 PMC3492448

[30] Kim D Jung J Lee Y Kwon HJ. Novel immunostimulatory phosphodiester oligodeoxynucleotides with CpT sequences instead of CpG motifs. Mol Immunol. (2011) 48:1494–504. 10.1016/j.molimm.2011.04.009 21529949

[31] Kim D Rhee JW Kwon S Sohn WJ Lee Y Kim DW. Immunostimulation and anti-DNA antibody production by backbone modified CpG-DNA. Biochem Biophys Res Commun. (2009) 379:362–7. 10.1016/j.bbrc.2008.12.063 19103173

[32] Ghosn EE Yang Y Tung J Herzenberg LA Herzenberg LA. CD11b expression distinguishes sequential stages of peritoneal B-1 development. Proc Natl Acad Sci U.S.A. (2008) 105:5195–200. 10.1073/pnas.0712350105 18375763 PMC2278228

[33] Ghosn EE Sadate-Ngatchou P Yang Y Herzenberg LA Herzenberg LA. Distinct progenitors for B-1 and B-2 cells are present in adult mouse spleen. Proc Natl Acad Sci USA. (2011) 108:2879–84. 10.1073/pnas.1019764108 21282663 PMC3041118

[34] Maharjan S Park BK Lee SI Lim Y Lee K Lee Y Kwon HJ. Gomisin G Suppresses the Growth of Colon Cancer Cells by Attenuation of AKT Phosphorylation and Arrest of Cell Cycle Progression. Biomol Ther. (2019) 27:210–15. 10.4062/biomolther.2018.054 29902863 PMC6430222

[35] Wu G Kim D Kim JN Park S Maharjan S Koh H. A Mucin1 C-terminal subunit-directed monoclonal antibody targets overexpressed mucin1 in breast cancer. Theranostics. (2018) 8:78–91. 10.7150/thno.21278 29290794 PMC5743461

[36] Hobson D Baker FA Curry RL Beare AS Massey PM. The efficacy of live and inactivated vaccines of Hong Kong influenza virus in an industrial community. A report to the Medical Research Council Committee on influenza and other respiratory virus vaccines. J Hyg (Lond). (1973) 71:641–7. 10.1017/s0022172400022907 4588772 PMC2130408

[37] Fukuyama S Kawaoka Y. The pathogenesis of influenza virus infections: the contributions of virus and host factors. Curr Opin Immunol. (2011) 23:481–6. 10.1016/j.coi.2011.07.016 21840185 PMC3163725

[38] Hutzler S Ozgor L Naito-Matsui Y Klasener K Winkler TH Reth M. The ligand-binding domain of Siglec-G is crucial for its selective inhibitory function on B1 cells. J Immunol. (2014) 192:5406–14. 10.4049/jimmunol.1302875 24790146

[39] Baumgarth N Waffarn EE Nguyen TT. Natural and induced B-1 cell immunity to infections raises questions of nature versus nurture. Ann N Y Acad Sci. (2015) 1362:188–99. 10.1111/nyas.12804 26060895 PMC4881423

[40] Choi YS Baumgarth N. Dual role for B-1a cells in immunity to influenza virus infection. J Exp Med. (2008) 205:3053–64. 10.1084/jem.20080979 19075288 PMC2605232

[41] Tripathi S Batra J Cao W Sharma K Patel JR Ranjan P. Influenza A virus nucleoprotein induces apoptosis in human airway epithelial cells: implications of a novel interaction between nucleoprotein and host protein Clusterin. Cell Death Dis. (2013) 4:e562. 10.1038/cddis.2013.89 23538443 PMC3615740

[42] Hinshaw VS Olsen CW Dybdahl-Sissoko N Evans D. Apoptosis: a mechanism of cell killing by influenza A and B viruses. J Virol. (1994) 68:3667–73 8189504 10.1128/jvi.68.6.3667-3673.1994 PMC236871

[43] Upham JP Pickett D Irimura T Anders EM Reading PC. Macrophage receptors for influenza A virus: role of the macrophage galactose-type lectin and mannose receptor in viral entry. J Virol. (2010) 84:3730–7. 10.1128/jvi.02148-09 20106926 PMC2849513

[44] Hashimoto Y Moki T Takizawa T Shiratsuchi A Nakanishi Y. Evidence for phagocytosis of influenza virus-infected, apoptotic cells by neutrophils and macrophages in mice. J Immunol. (2007) 178:2448–57. 10.4049/jimmunol.178.4.2448 17277152

[45] Tate MD Ioannidis LJ Croker B Brown LE Brooks AG Reading PC. The role of neutrophils during mild and severe influenza virus infections of mice. PLoS ONE. (2011) 6:e17618. 10.1371/journal.pone.0017618 21423798 PMC3056712

[46] Weis W Brown JH Cusack S Paulson JC Skehel JJ Wiley DC. Structure of the influenza virus haemagglutinin complexed with its receptor, sialic acid. Nature. (1988) 333:426–31. 10.1038/333426a0 3374584

[47] Yu WC Chan RW Wang J Travanty EA Nicholls JM Peiris JS. Viral replication and innate host responses in primary human alveolar epithelial cells and alveolar macrophages infected with influenza H5N1 and H1N1 viruses. J Virol. (2011) 85:6844–55. 10.1128/jvi.02200-10 21543489 PMC3126566

[48] Song Y Wang X Zhang H Tang X Li M Yao J. Repeated low-dose influenza virus infection causes severe disease in mice: a model for vaccine evaluation. J Virol. (2015) 89:7841–51. 10.1128/jvi.00976-15 25995265 PMC4505666

[49] Fang Y Banner D Kelvin AA Huang SS Paige CJ Corfe SA. Seasonal H1N1 influenza virus infection induces cross-protective pandemic H1N1 virus immunity through a CD8-independent, B cell-dependent mechanism. J Virol. (2012) 86:2229–38. 10.1128/jvi.05540-11 22130540 PMC3302411

[50] Alsharifi M Furuya Y Bowden TR Lobigs M Koskinen A Regner M. Intranasal flu vaccine protective against seasonal and H5N1 avian influenza infections. PLoS ONE. (2009) 4:e5336. 10.1371/journal.pone.0005336 19401775 PMC2671162

[51] Groves HT McDonald JU Langat P Kinnear E Kellam P McCauley J. Mouse models of influenza infection with circulating strains to test seasonal vaccine efficacy. Front Immunol. (2018) 9:126. 10.3389/fimmu.2018.00126 29445377 PMC5797846

[52] Thangavel RR Bouvier NM. Animal models for influenza virus pathogenesis, transmission, and immunology. J Immunol Methods. (2014) 410:60–79. 10.1016/j.jim.2014.03.023 24709389 PMC4163064

[53] Edenborough KM Gilbertson BP Brown LE. A mouse model for the study of contact-dependent transmission of influenza A virus and the factors that govern transmissibility. J Virol. (2012) 86:12544–51. 10.1128/jvi.00859-12 22951824 PMC3497676

[54] Iwatsuki-Horimoto K Nakajima N Ichiko Y Sakai-Tagawa Y Noda T Hasegawa H. Syrian hamster as an animal model for the study of human influenza virus infection. J Virol. (2018) 92 10.1128/jvi.01693-17 29212926 PMC5790951

[55] Armstrong SM Wang C Tigdi J Si X Dumpit C Charles S. Influenza infects lung microvascular endothelium leading to microvascular leak: role of apoptosis and claudin-5. PLoS ONE. (2012) 7:e47323. 10.1371/journal.pone.0047323 23115643 PMC3480371

[56] Wang C Armstrong SM Sugiyama MG Tabuchi A Krauszman A Kuebler WM. Influenza-induced priming and leak of human lung microvascular endothelium upon exposure to *Staphylococcus aureus*. Am J Respir Cell Mol Biol. (2015) 53:459–70. 10.1165/rcmb.2014-0373oc 25693001

[57] Christensen JP Doherty PC Branum KC Riberdy JM. Profound protection against respiratory challenge with a lethal H7N7 influenza A virus by increasing the magnitude of CD8(+) T-cell memory. J Virol. (2000) 74:11690–6. 10.1128/jvi.74.24.11690-11696.2000 11090168 PMC112451

[58] Williams MA Bevan MJ. Effector and memory CTL differentiation. Annu Rev Immunol. (2007) 25:171–92. 10.1146/annurev.immunol.25.022106.141548 17129182

