# Supplementary material for: Peritoneal Cells Mediate Immune Responses and Cross-Protection Against Influenza A Virus
Source: Front Immunol. 2019 May 28;10:1160. doi: 10.3389/fimmu.2019.01160 (PMC6546726; doi:10.3389/fimmu.2019.01160)
Supplement: Supplementary file 1 [file Presentation_1.pdf]

Supplementary Material

Peritoneal Cells Mediate Immune Responses and Cross-Protection Against Influenza A Virus

Avishekh Gautam, Byoung Kwon Park, Te Ha Kim, Madhav Akauliya, Dongbum Kim, Sony Maharjan, Joongwon Park, Jinsoo Kim, Hanseul Lee, Man-Seong Park, Younghee Lee*, Hyung-Joo Kwon*

*** Correspondence:** Younghee Lee: yhl4177@cbnu.ac.kr, Hyung-Joo Kwon: hjookwon@hallym.ac.kr

Supplementary Methods

Mice and Infection

Eight-week-old BALB/c (H-2^b) mice were maintained under SPF conditions. Infection was performed intraperitoneally with influenza A virus (A/WSN/1933) at selected pfu per mouse under animal biosafety level 2 condition. After 14 days, the production of A/WSN/1933 virus-reactive IgG was determined by ELISA using 96-well immunoplates (Nunc^{TM}, Roskilde, Denmark) coated with A/WSN/1933 virus.

Annexin V Staining

The mouse macrophage cell line, RAW 264.7, was maintained in complete medium-specifically, Dulbecco's modified Eagle's medium (DMEM), containing 1% penicillin-streptomycin and 10% fetal bovine serum. RAW 264.7 cells were seeded into 24-well plates (Nunc^{TM}, Denmark) at a density of 1 x 10^6 cells per well. A/WSN/1933 virus or UV-inactivated A/WSN/1933 (UV-WSN) viruses and/or SNA (10 µg/ml) were added to each well at an MOI of 1. After incubation at 37 °C for 72 h, the cells were harvested and stained with APC-conjugated Annexin V (eBioscience, San Diego, CA, USA) for 15 min at room temperature. Splenocytes were prepared from BALB/c mice and plated in24-well plates at a density of 1 x 10^6 cells per well. A/WSN/1933 virus (1 x 10^6 pfu) or SNA (10 µg/ml) was added to each well. After incubation at 37 °C for 72 h, the cells were harvested and stained with anti-FcγRII/III (Fc receptor blocker) before being stained with APCeF780-conjugated anti-CD19, FITC-conjugated anti-B220, and PerCP Cy5.5-conjugated anti-CD3 antibodies (BD Biosciences) for 1 h. The cells were again stained with APC-conjugated Annexin V (eBioscience, San Diego, CA, USA) for 15 min at room temperature. To exclude dead cells, stained cells were incubated with 10 µg/mL of PI (eBioscience, San Diego, CA, USA). The cells were analyzed with a FACSCanto II (Becton Dickinson).

Lectin Staining

Biotinylated *Maackia amurensis* lectin II (MAL II) and fluorescein-conjugated SNA were obtained from Vector Laboratories (Burlingame, CA, USA). Peritoneal cells and splenocytes were harvested from BALB/c mice. The cells were stained with anti-FcγRII/III (Fc receptor blocker) before being

stained with APCeF780-conjugated anti-CD19, FITC-conjugated anti-B220, FITC-conjugated anti-F4/80, BV421-conjugated anti-CD11b antibodies (BD Biosciences) for 1 h at 4 °C. For the detection of human influenza virus-specific α-2,6-linked sialic acids, cells were stained with fluorescein-conjugated SNA. For avian influenza virus-specific α-2,3-linked sialic acids, peritoneal cells were stained with biotinylated MAL II followed by APC-conjugated streptavidin (Thermo Fisher Scientific). After 1 h of incubation at 4 °C, the cells were analyzed by flow cytometry (BD FACSCalibur™, BD Biosciences).

Virus Challenge Experiments

Eight-week-old BALB/c (H-2^b) mice (n=10) were injected intraperitoneally with wt A/Hong Kong/4801/2014 (H3N2) virus at the indicated pfu per mouse. The mice were observed for 8 days to monitor clinical signs and body weight.

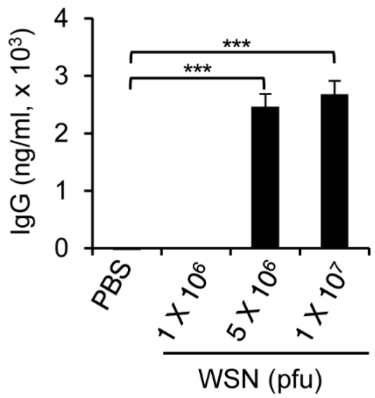

Supplementary Figure S1. Antibody production following intraperitoneal infection with A/WSN/1933 virus. BALB/c mice (n=5/group) were challenged intraperitoneally with the indicated pfu of A/WSN/1933 virus per mouse and anesthetized after 14 days. The amount of virus-reactive IgG in the peritoneal cavity fluids was measured by ELISA. ***p < 0.0005.

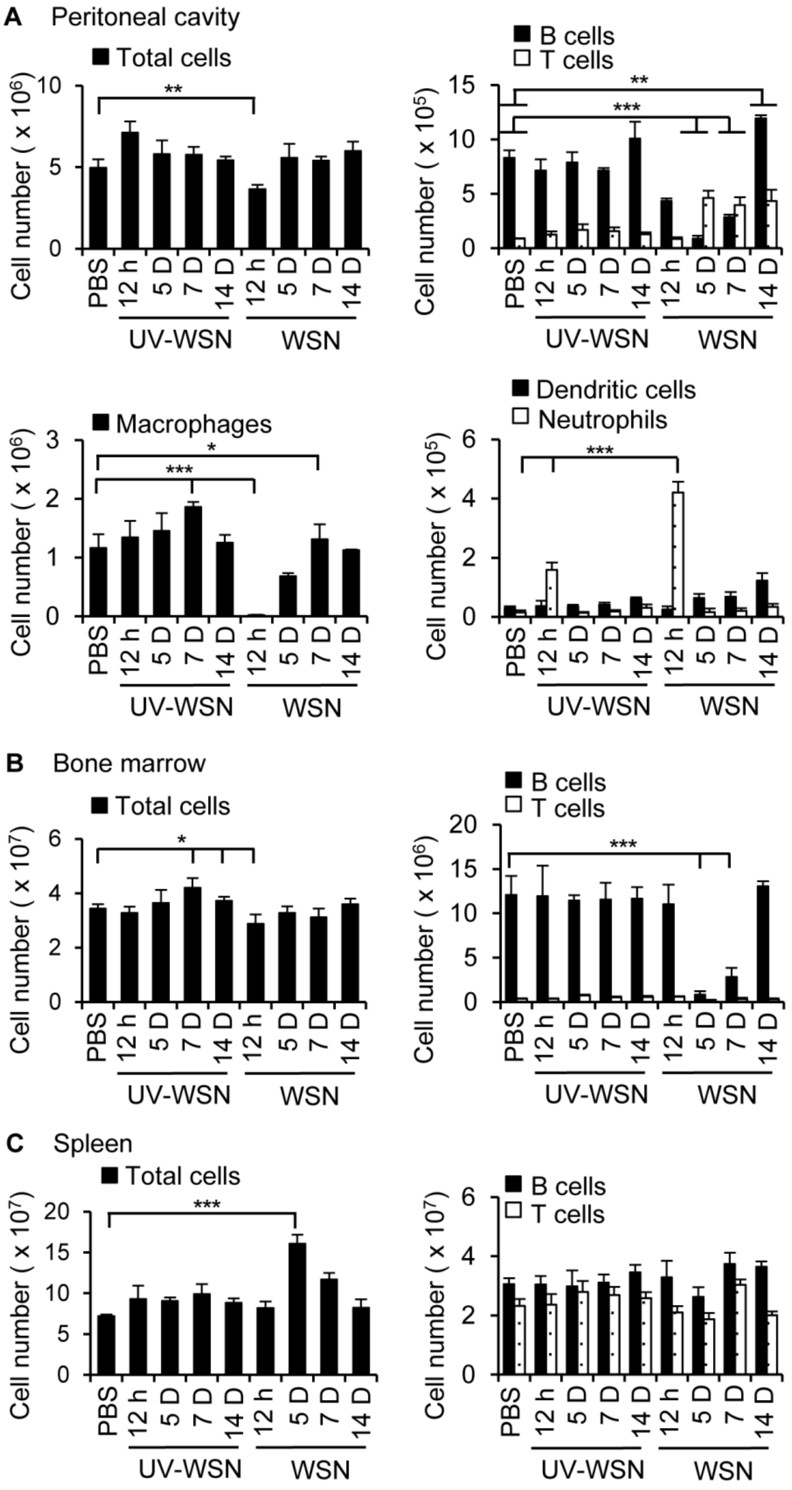

Supplementary Figure S2. (Corresponding to Figure 2) Analysis of cell numbers following intraperitoneal challenge with A/WSN/1933 virus or UV-inactivated A/WSN/1933 virus. BALB/c mice (n=5/group) were sacrificed at 12 h, 5 days, 7 days, and 14 days after intraperitoneal challenge with 5 x 10^6 pfu of A/WSN/1933 virus (WSN) or UV-inactivated A/WSN/1933 virus (UV-WSN). Peritoneal cells, bone marrow cells and splenocytes were harvested, counted, and stained with fluorescence-conjugated antibodies and analyzed by flow cytometry. Cells from control mice at 14 days after intraperitoneal injection of PBS were used as an uninfected control. **(A)** Number of total

cells, B cells, T cells, macrophages, dendritic cells, and neutrophils of the peritoneal cavity/mouse. **(B)** Number of total cells, B cells, and T cells of the bone marrow/mouse. **(C)** Number of total cells, B cells, and T cells of the spleen/mouse. Each figure is representative of three independent experiments. $*p < 0.05$, $**p < 0.005$, $***p < 0.0005$.

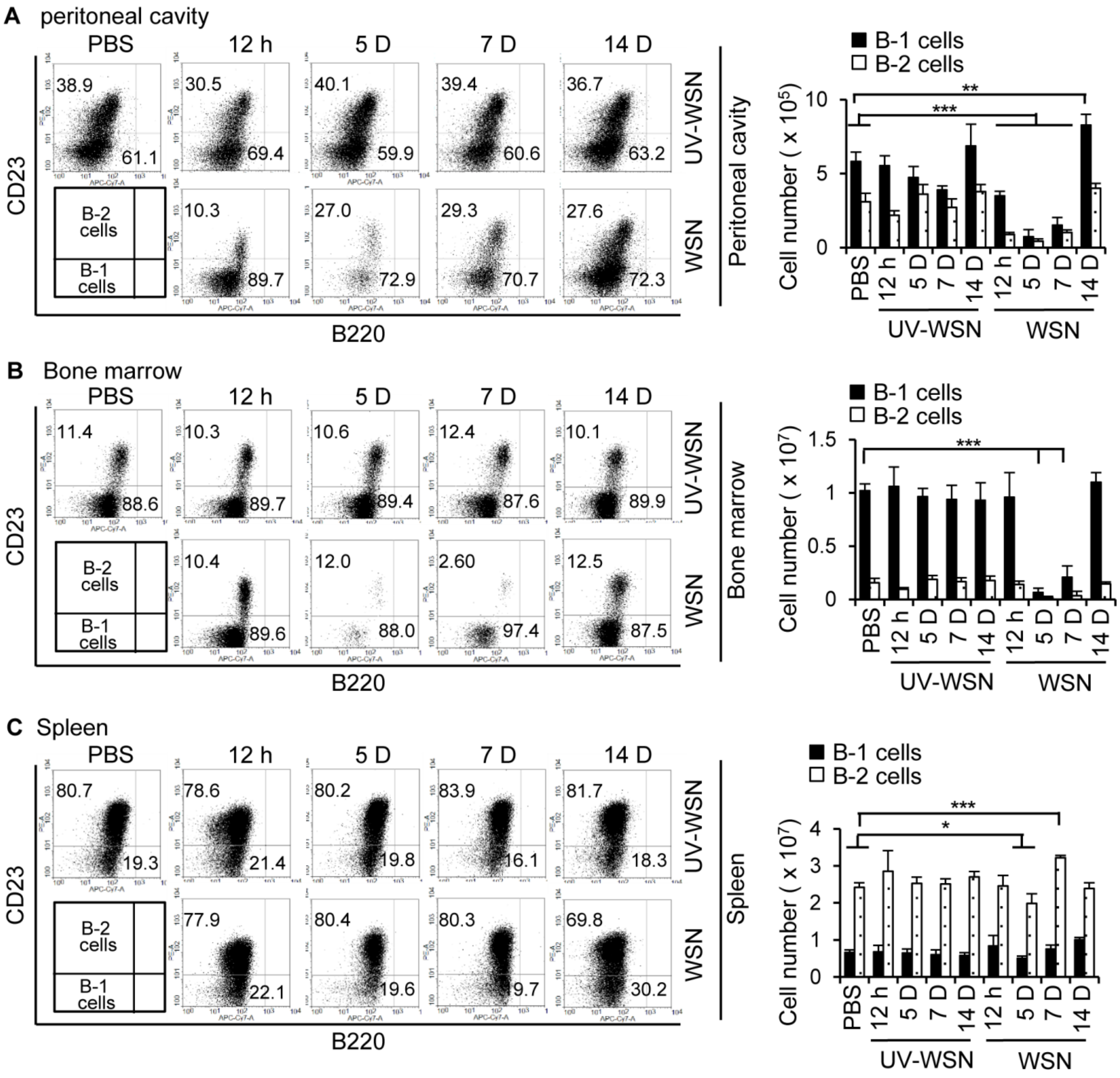

Supplementary Figure S3. (Corresponding to Figure 2) Analysis of B cells following intraperitoneal challenge with A/WSN/1933 virus or UV-inactivated A/WSN/1933 virus. BALB/c mice (n=5/group) were sacrificed at 12 h, 5 days, 7 days, and 14 days after intraperitoneal challenge with 5 x 10^6 pfu of A/WSN/1933 virus (WSN) or UV-inactivated A/WSN/1933 virus (UV-WSN). Peritoneal cells, bone marrow cells and splenocytes were harvested, counted, and stained with fluorescence-conjugated antibodies and analyzed by flow cytometry. Cells from control mice at 14 days after intraperitoneal injection of PBS were used as an uninfected control. **(A-C)** $FSC^{low}SSC^{low}$ cells of peritoneal cells, bone marrow cells, splenocytes were gated, and lymphocyte populations of the peritoneal cavity **(A)**,

bone marrow **(B)**, and spleen **(C)** were gated using CD19 and sorted into B220 and CD23 subsets. **(A)** Populations of B-1 cells and B-2 cells of the peritoneal cavity (left panel). Number of B-1 cells and B-2 cells of the peritoneal cavity/mouse (right panel). **(B)** Populations of B-1 cells and B-2 cells of the bone marrow (left panel). Number of B-1 cells and B-2 cells of the bone marrow/mouse (right panel). **(C)** Populations of B-1 cells and B-2 cells of the spleen (left panel). Number of B-1 cells and B-2 cells of the spleen/mouse (right panel). Each figure is representative of three independent experiments. **p* < 0.05, ***p* < 0.005, ****p* < 0.0005.

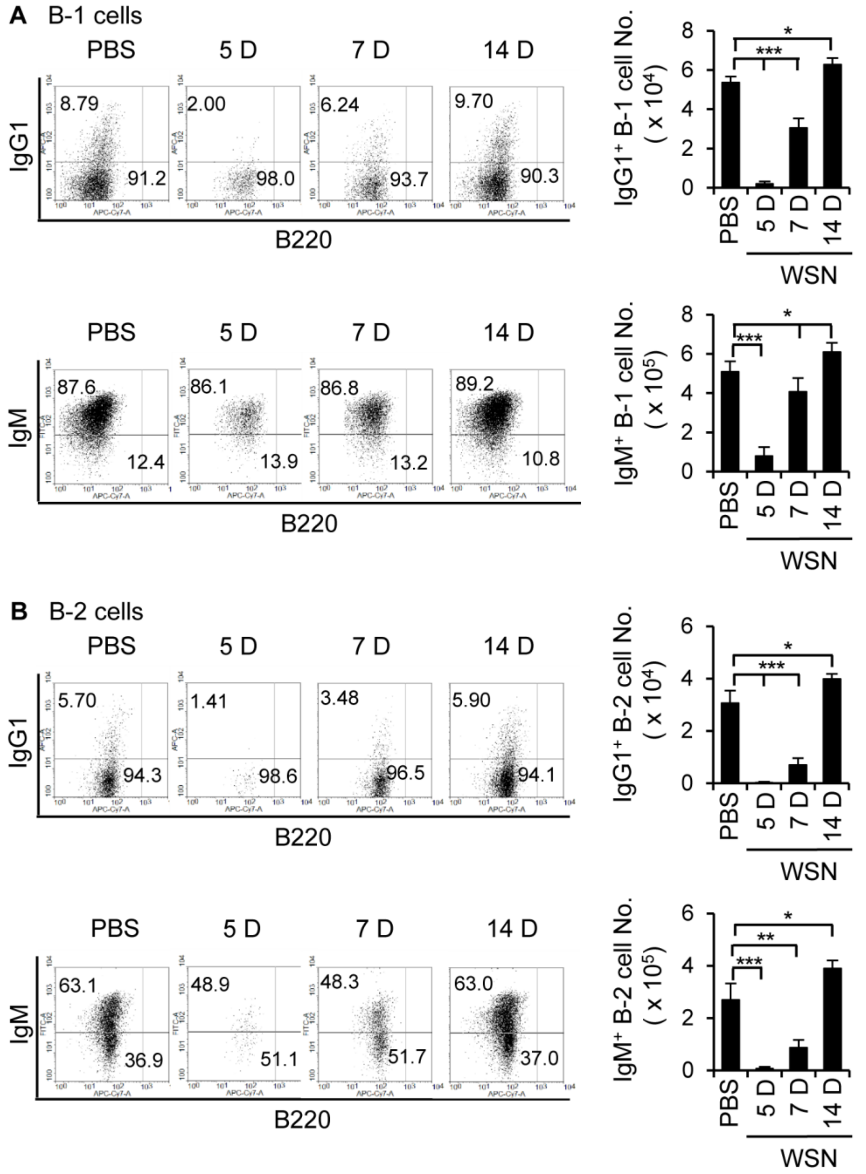

Supplementary Figure S4. Analysis of IgG+ or IgM+ subsets following intraperitoneal challenge with A/WSN/1933 virus. BALB/c mice (n=5/group) were sacrificed at 5 days, 7 days, and 14 days after intraperitoneal challenge with 5 x 10^6 pfu of A/WSN/1933 virus (WSN). Peritoneal cells were harvested, counted, and stained with fluorescence-conjugated antibodies and analyzed by flow cytometry. Cells from control mice at 14 days after intraperitoneal injection of PBS were used as an

uninfected control. **(A)** Populations of B-1 cells. FSC^{low}SSC^{low} cells of peritoneal cells were gated using CD19, and then sorted into B220 and CD23 subsets. **(A)** Populations of IgG1^{+} or IgM^{+} B-1 cells of the peritoneal cavity. **(B)** Populations of IgG1^{+} or IgM^{+} B-2 cells of the peritoneal cavity. Right panel showed the number of IgG1^{+} B-1 cells, IgM^{+} B-1 cells, IgG1^{+} B-2 cells, or IgM^{+} B-2 cells of the peritoneal cavity/mouse.

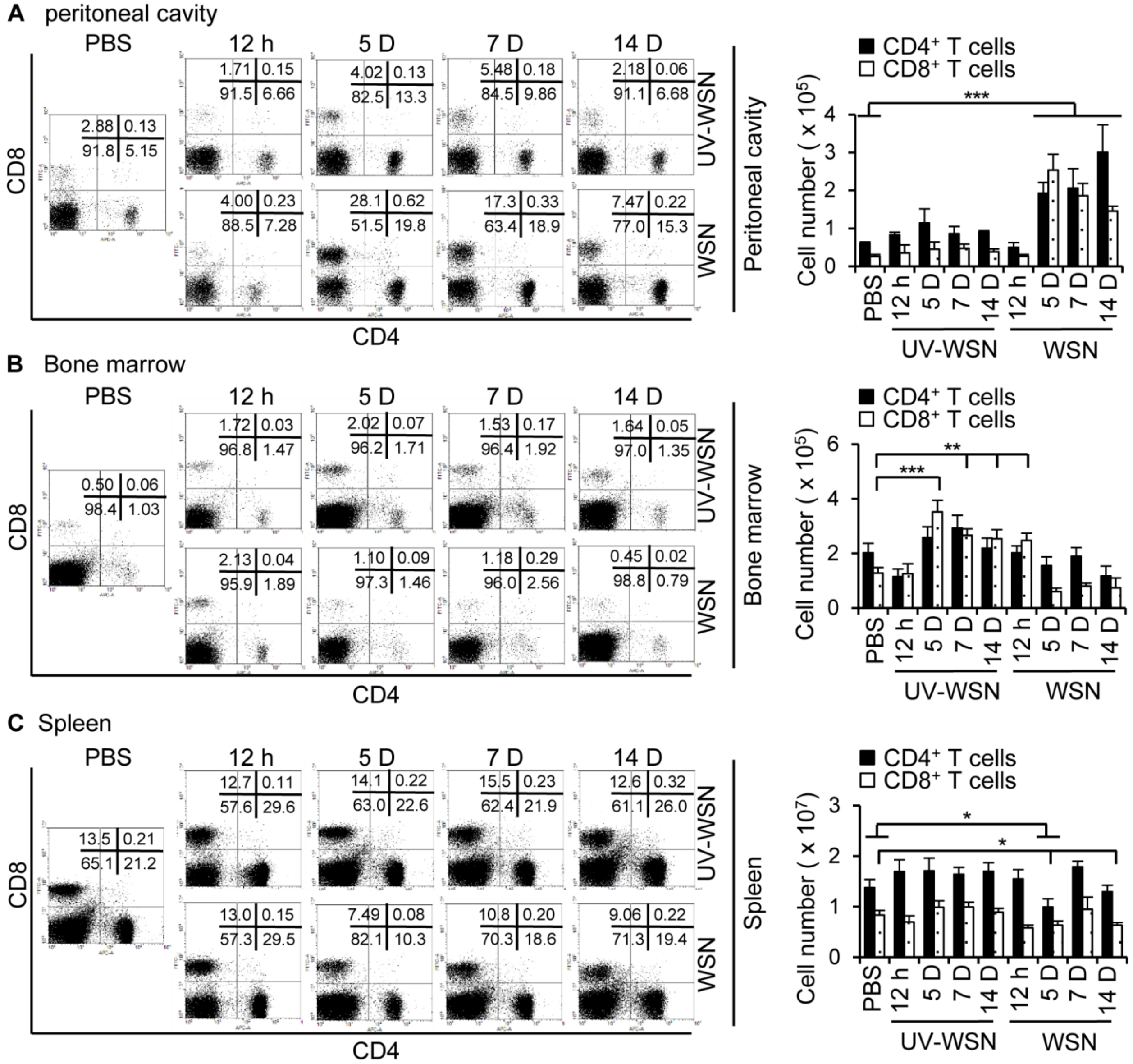

Supplementary Figure S5. (Corresponding to Figure 2) Analysis of T cells following intraperitoneal challenge with A/WSN/1933 virus or UV-inactivated A/WSN/1933 virus. BALB/c mice (n=5/group) were sacrificed at 12 h, 5 days, 7 days, and 14 days after intraperitoneal challenge with 5 x 10^{6} pfu of A/WSN/1933 virus (WSN) or UV-inactivated A/WSN/1933 virus (UV-WSN). Peritoneal cells, bone marrow cells and splenocytes were harvested, counted, and stained with fluorescence-conjugated antibodies and analyzed by flow cytometry. Cells from control mice at 14 days after intraperitoneal injection of PBS were used as an uninfected control. **(A-C)** FSC^{low}SSC^{low} cells of peritoneal cells,

bone marrow cells, splenocytes were gated, and lymphocyte populations of the peritoneal cavity **(A)**, bone marrow **(B)**, and spleen **(C)** were sorted into CD4 and CD8 subsets. **(A)** Populations of $CD4^+$ T cells and $CD8^+$ T cells of the peritoneal cavity (left panel). Number of $CD4^+$ T cells and $CD8^+$ T cells of the peritoneal cavity/mouse (right panel). **(B)** Populations of $CD4^+$ T cells and $CD8^+$ T cells of the bone marrow (left panel). Number of $CD4^+$ T cells and $CD8^+$ T cells of the bone marrow/mouse (right panel). **(C)** Populations of $CD4^+$ T cells and $CD8^+$ T cells of the spleen (left panel). Number of $CD4^+$ T cells and $CD8^+$ T cells of the spleen/mouse (right panel). Each figure is representative of three independent experiments. $*p < 0.05$, $**p < 0.005$, $***p < 0.0005$.

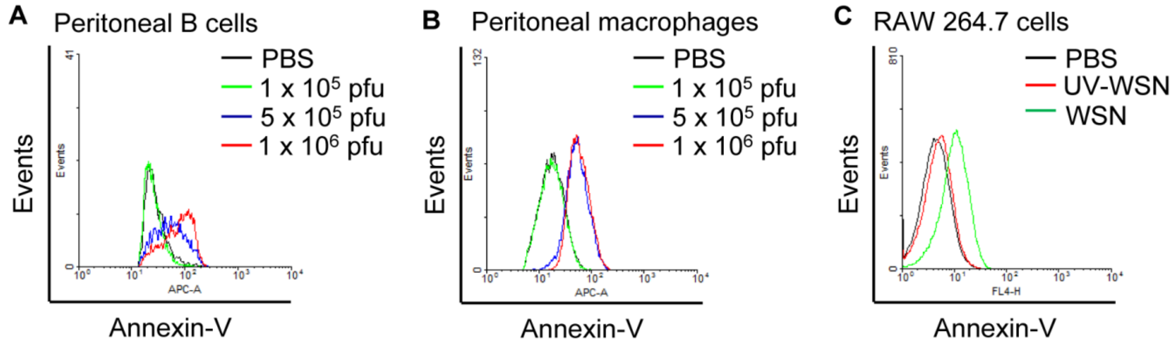

Supplementary Figure S6. Apoptosis of peritoneal cells and RAW 264.7 cells after A/WSN/1933 virus infection. **(A, B)** Peritoneal cells (1 x 10^6 cells per well) were cultured in 24-well plates, and A/WSN/1933 virus at the indicated pfu was added to each well. After 72 h of incubation, cells were collected, stained with fluorescence-conjugated antibodies (CD19 and B220 for lymphoid cells, CD11b and F4/80 for myeloid cells) and APC-conjugated Annexin V, and then analyzed by flow cytometry. **(C)** RAW 264.7 cells were treated with A/WSN/1933 virus or UV-WSN virus for 72 h, followed by staining with APC-conjugated Annexin V and analysis by flow cytometry. Annexin V-positive RAW 264.7 cells are represented in histograms.

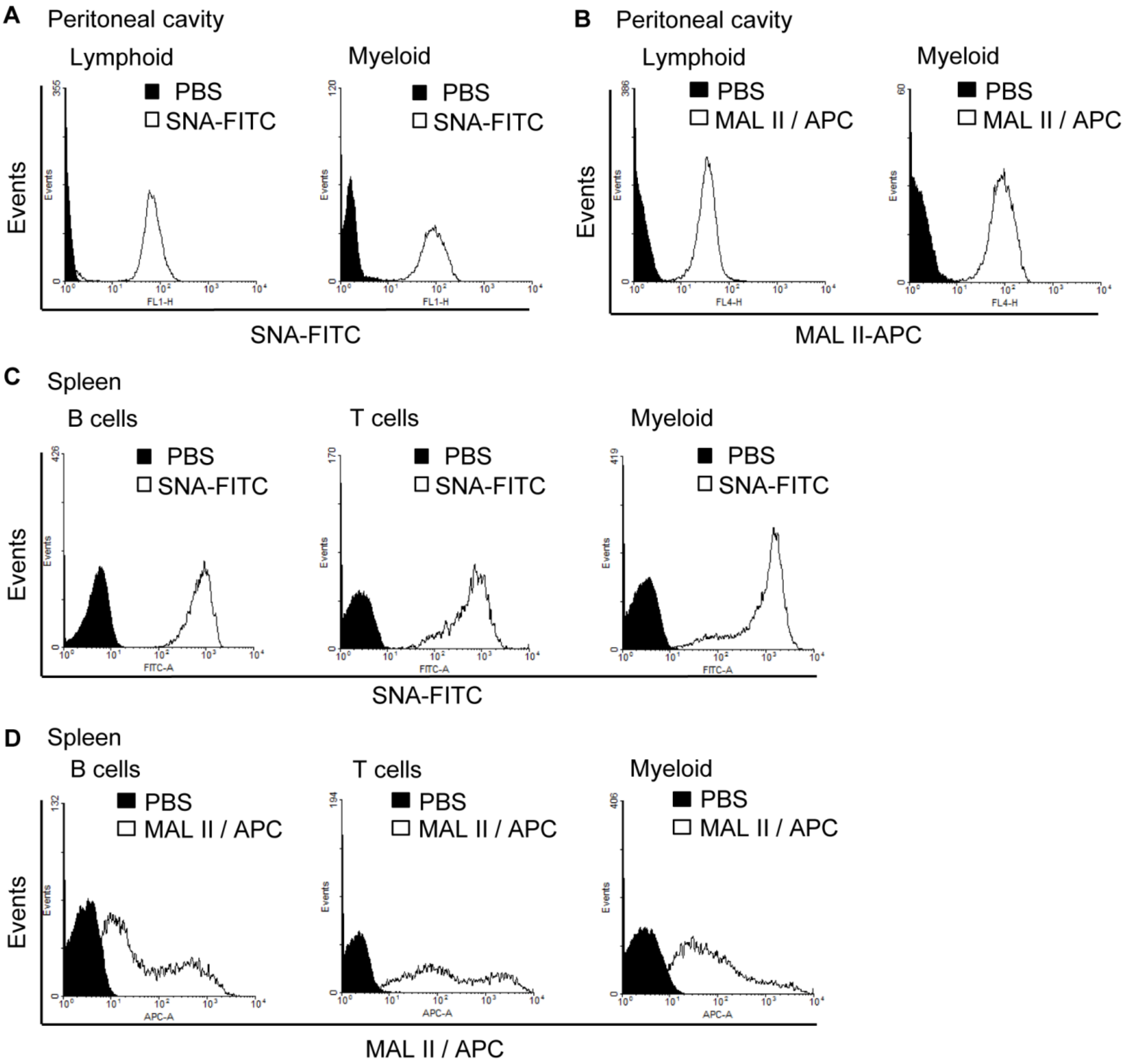

Supplementary Figure S7. Detection of virus-specific sialic acids in peritoneal cells and splenocytes. Peritoneal cells and splenocytes were harvested from BALB/c mice and stained with fluorescein-conjugated SNA or biotinylated MAL II followed by treatment with APC-conjugated streptavidin. **(A) (B)** The binding of SNA **(A)** and MAL II **(B)** to the peritoneal lymphoid and myeloid cells was determined by flow cytometry. **(C, D)** The binding of SNA **(C)** and MAL II **(D)** to the splenic B cells, T cells and myeloid cells was determined by flow cytometry.

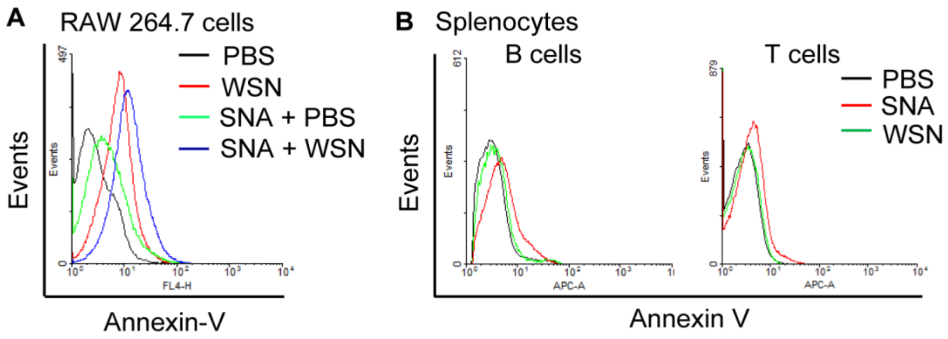

Supplementary Figure S8. A/WSN/1933 virus-reactive apoptosis of RAW 264.7 cells. **(A)** RAW 264.7 cells were incubated in 24-well plates with SNA for 1 h at 37 °C and then treated with PBS (SNA+PBS) or 1 x 10^6 pfu of A/WSN/1933 virus (SNA+WSN) for 72 h. **(B)** Splenocytes were incubated in 24-well plates with PBS, SNA, or 1 x 10^6 pfu of A/WSN/1933 virus (WSN) for 72 h. After 72 h of incubation, the cells were stained with fluorescence-conjugated antibodies (CD19, B220; left) or fluorescence-conjugated antibodies (CD3; right) and then stained with Annexin V. Annexin V-positive B cells and T cells were analyzed by flow cytometry and represented in histograms. All experiments were performed in triplicate.

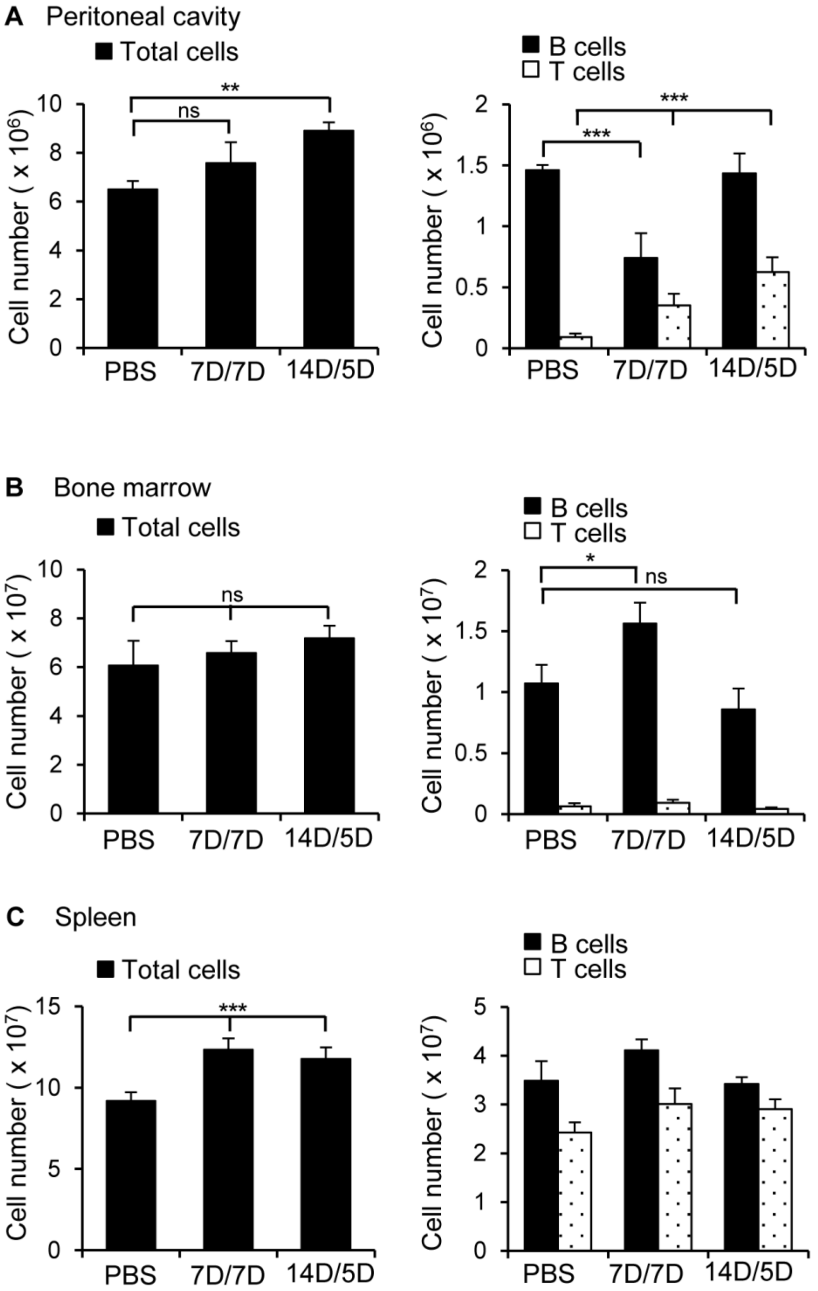

Supplementary Figure S9. (Corresponding to Figure 5) Re-infection with A/WSN/1933 virus limits B cell death and induces antibody production. BALB/c mice (n=5/group) were sacrificed after repeated intraperitoneal inoculation with PBS or 5 x 10^6 pfu of A/WSN/1933 virus. 7D/7D represents a procedure consisting of a first infection, a second infection after 7 days, and then sacrifice after another 7 days. 14D/5D represents a procedure of first infection, second infection after 14 days, and then sacrifice after another 5 days. **(A)** Number of total cells, B cells, and T cells of the peritoneal cavity/mouse. **(B)** Number of total cells, B cells, T cells of the bone marrow/mouse. **(C)** Number of total cells, B cells, T cells, of the spleen/mouse. Each figure is representative of three independent experiments. $*p < 0.05$, $**p < 0.005$, $***p < 0.0005$.

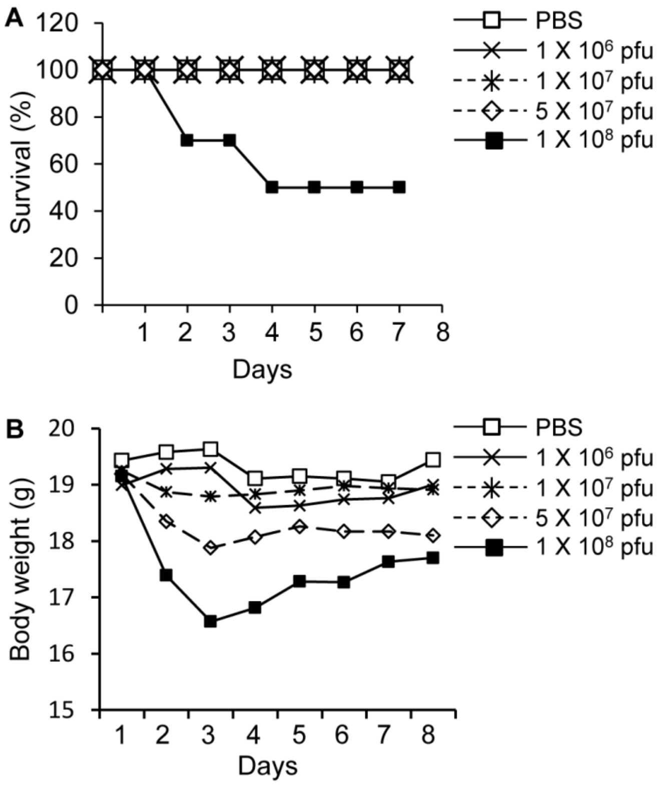

Supplementary Figure S10. Effect of wt A/Hong Kong/4801/2014 (H3N2) virus challenge on mice survival. BALB/c mice (n=10/group) were challenged intraperitoneally with wt A/Hong Kong/4801/2014 (H3N2) virus at the indicated pfu per mouse. The survival **(A)** and the body weight **(B)** of the challenged mice were recorded for 8 days.
